# Molecular bases for drought tolerance in common vetch: designing new molecular breeding tools

**DOI:** 10.1186/s12870-020-2267-z

**Published:** 2020-02-13

**Authors:** Lucía De la Rosa, Encarnación Zambrana, Elena Ramirez-Parra

**Affiliations:** 1 Centro Nacional de Recursos Fitogenéticos, Instituto Nacional de Investigación y Tecnología Agraria y Alimentaria, 28800 Alcalá de Henares, Spain; 2 Centro de Biotecnología y Genómica de Plantas, Instituto Nacional de Investigación y Tecnología Agraria y Alimentaria, Universidad Politécnica de Madrid, Campus de Montegancedo, 28223 Pozuelo de Alarcón, Spain

**Keywords:** Common vetch (*Vicia sativa* L.), Drought response, Genetic variation, Molecular breeding, Gene expression

## Abstract

**Background:**

Common vetch (*Vicia sativa* L.) is a forage grain legume of high protein content and high nitrogen fixation, relevant in sustainable agriculture systems. Drought is the main limiting factor of this crop yield. Genetic resources collections are essential to provide genetic variability for breeding. The analysis of drought associated parameters has allowed us to identify drought tolerant and sensitive ecotypes in a vetch core collection.

**Results:**

To understand the mechanisms involved in drought response we analysed transcriptomic differences between tolerant and sensitive accessions. Polymorphic variants (SNPs and SSRs) in these differential expressed genes (DEGs) have also been analysed for the design of drought-associated markers**.** A total of 1332 transcripts were commonly deregulated in both genotypes under drought. To know the drought adaptive response, we also analysed DEGs between accessions. A total of 2646 transcripts are DEG between sensitive and tolerant ecotypes, in watered and drought conditions, including important genes involved in redox homeostasis, cell wall modifications and stress-response. The integration of this functional and genetic information will contribute to understand the molecular mechanisms of drought response and the adaptive mechanisms of drought tolerance in common vetch. The identification of polymorphic variants in these DEGs has also been screened for the design of drought-associated markers that could be used in future breeding program strategies*.*

**Conclusions:**

Our studies shed light for the first time in common vetch about the genes and pathways associated with drought tolerance. In addition, we identify over 100 potential drought associated polymorphism, as SNPs or SSRs, which are differently present in drought and tolerant genotypes. The use of these molecular markers for trait prediction would enable the development of genomic tools for future engineering strategies by screening of germplasm crop collections for traits related with crop drought resilience, adaptability or yield in vetch.

## Background

Drought causes large losses in the crop production in the arid and semiarid areas of the planet. FAO believes that stress by water deficit is a major cause of food shortages in developing countries, significantly surpassing other types of environmental threats, and it is believed that the result of global warming will increase the frequency and impact of this phenomenon (http://www.fao.org/).

Drought response includes a plethora of morphological, physiological, biochemical and molecular changes both at the whole plant and at cellular levels. Primary drought response entails reduction of relative water content and turgor alteration that activates stomatal closure, reduces transpiration and limits the photosynthesis rate. Important alterations are also produced at radicular level where root architecture and physiology are affected to regulate homeostasis of water uptake to maintain osmotic pressure [1]. The more relevant aspects of plant responses to drought involve maintenance of osmotic and oxidative homeostasis. Drought promotes the accumulation of Reactive Oxygen Species (ROS) overcoming the antioxidant capacity of cells and inducing changes in the activities of antioxidant enzymes to increase the scavenging capacity against ROS [2, 3]. These alterations promote drastic modifications in the cellular redox homeostasis that may stimulate the damage of proteins, lipids and DNA [4]. Accumulation of osmolytes also plays a key role in adaptation to water deficit [3]. The regulatory factors, such as transcription factors and protein kinases, play important roles in improving plant tolerance to drought and other abiotic stresses [5].

To cope with drought, plants have developed adaptive strategies to tolerate these stress conditions. These modifications involve physiological and biochemical alterations linked to the function of many stress-associated genes. Although many of these responses are common for most plant species, some drought responses present species- and even genotype-specific characteristics [6, 7]. This fact increases the diversity and complexity to understand the mechanisms of drought tolerance.

The common vetch (*Vicia sativa L.)* is a legume of double use, as forage and grain for animal feed, with high protein content and great capacity of biological fixation of nitrogen [8], thus has an enormous potential in systems of sustainable agriculture (www.fao.org). As cover crop, besides enriching the soil with nitrogen, it prevents the growth of other plants that compete for soil nutrients, reducing the use of herbicides [9, 10]. The enormous dependence of proteins of vegetable origin for animal feed and the interest in the European Union and other world regions for the use of species of environmental value or “greening” makes the use of common vetch an agricultural alternative of great economic importance. The Spanish Plant Genetic Resources Center (CRF) belonging to the National Institute for Agricultural and Food Research and Technology (INIA) preserves the national active collection of *V. sativa L.* with over 800 accessions including landraces, wild relatives and commercial cultivars from worldwide origin. The agromorphological results, available at CRF website (http://wwwx.inia.es/coleccionescrf/CaracterizacionCRFeng.asp) and the analysis of the genetic diversity of the collection from seed reserve protein studies, were a first step that allowed us to rationalise the collection by analysing the available information and establishing a preliminary core collection of 53 entries [11, 12].

Environmental stresses, as drought, severely constrain common vetch production; therefore, identification of drought tolerant genotypes is an essential target in breeding programs. Based on the previous selection of accessions from our collection with traits of drought tolerance [13], in this work we performed a transcriptomic analysis under irrigation and drought conditions, to identify drought tolerant candidate genes.

Next-generation sequencing platforms for RNA-sequencing (RNA-Seq) has been used to analyse the genetic basis of abiotic stress responses in plants, especially in non-model species for which genomic resources are unavailable [14]. These tools have allowed a preliminary exploration of the gene expression of in some vetch tissues [15, 16]. Here, de novo transcriptome assembly, dataset gene annotation and analysis of differential gene expression have allowed us to identify not only drought regulated genes but also, and most relevant, genes that are expressed differentially between tolerant and sensitive genotypes. These transcripts include genes involved in general stress response, cell wall organization, water deprivation, oxidative stress and abscisic acid (ABA) response genes, which are differentially expressed between tolerant and sensitive variants. In addition, these studies are allowing us to understand the mechanisms of stress response and tolerance to water deficit.

Moreover, our transcriptomic analyses will contribute to the development of genomic tools for drought tolerance prediction, since we have identified variants, such single nucleotide polymorphisms (SNPs) and simple sequence repeats (SSRs) on DEG genes. The integration of this functional and genetic information could be used in future strategies to accelerate breeding programs and in the development of predictive genotypic and phenotypic analysis for further use in gene-banks.

## Results

### Characterization of drought-tolerance traits

**Fig. 1:**
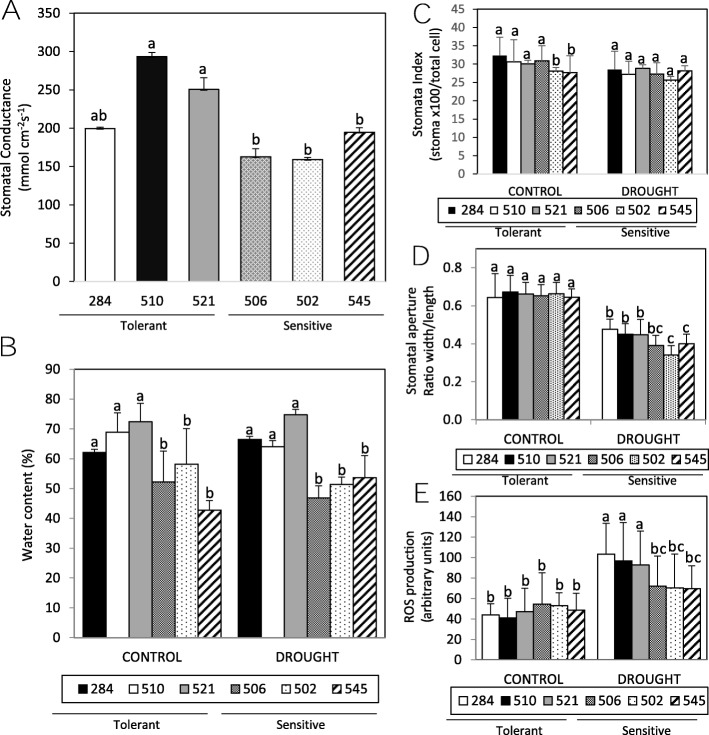
Differences in stomatal conductance, transpiration water loss and stomatal dynamics for accession identified as drought tolerant (284, 510 and 521 identified with plain colours on the histograms bars of all the figures) and drought sensitives (502, 506 and 545 identified with ornamented wefts on the histogram bars of all the figures). **a.** Stomatal conductance values measured in the field. Graphic indicates a representative measurement (May 2018; rainfed condition; “La Canaleja” Madrid), recorded in triplicate on at least 6 plants of each variety. **b.** Water content and transpiration water loss from detached vetch leaflets of greenhouse-grown plants 3 h after detach. One-week-old vetch varieties were grown with (control) or without water (drought) for additional 3 weeks, before being dried and weight at 3 h. Relative leaf weight was expressed as a percentage based upon the initial fresh weight (% FW). Three independent experiments; *n* = 20 leaflets each. Statistical differences (ANOVA and Tukey HSD test) are only referred to same time, for simplification. **c.** Analysis of stomatal index (number of stomatal complex×100/number of total epidermal cells). Cell analysis of abaxial leaf epidermal layers (4-week -old leaves) of different greenhouse-grown genotypes analysed by microscopy. **d.** Stomatal closure by drought. Stomatal apertures were measured on epidermal peels of leaves from greenhouse-grown 4-week-old plants grown in control (white columns) or drought (black columns) conditions. Values obtained from triplicate experiments (*n* > 60). **e.** ROS production was analysed by measuring carboxifluorescein diacetate fluorescence levels in guard cells from greenhouse-grown 4-week-old plants grown in control (white columns) or drought (black columns) conditions. Experiments were carried out in triplicate. **a-e.** Values are means ± sd. Different letters indicate significantly differences analysed by ANOVA and Tukey HSD post hoc test post-test; *p* < 0.05

Drought stress causes numerous molecular, biochemical and physiological changes. One of the early symptoms of water deficiency in plants is the decrease of Relative Water Content under drought stress conditions [17]. Stomatal conductance is one parameter directly related to tolerance to water deficit stress. Stomatal conductance measures under different meteorological conditions were analysed, indicating higher levels in accessions 284, 510, and 521, previously identified as tolerant genotypes (Fig. 1a). Decrease in leaf evapotranspiration is an essential mechanism to achieve a better adaptation to the drought. A transpiration assay was carried out by measuring the water loss in leaflets detached from common vetch plants. We found that water content decreased more quickly in accessions 284, 510 and 521, previously selected as drought tolerant candidates (Fig. 1b). Decreased of water content could be explained by reduction in stomatal index or stomatal aperture. The analysis of stomata number, density of guard cells and stomatal index do not showed statistical differences (Fig. 1c). During drought stress, ABA promotes stomatal closing that minimises water loss by transpiration [18, 19]. Our analyses indicate a general stomatal closure in drought conditions, as expected. Although no drastic differences were found between genotypes, sensitive candidates 506, 502 and 545 shown slightly major closure in drought conditions than tolerant accessions (Fig. 1d). Reactive Oxygen Species (ROS) is a secondary messenger that is commonly triggered in response to diverse stimuli, modulating ABA-induced closure of guard cells [18, 19]. Stomatal carboxyfluorescein staining indicates a general increase of ROS production in plants after drought, especially in the drought tolerant candidates (Fig. 1e). These data suggest that stress tolerance in vetch could be due to reduced transpiration rate mediated by enhanced stomatal closure and an alteration in ROS production dynamic in guard cells.

The levels of epicuticular waxes and protectant osmolytes (including proline and soluble sugars) may affect drought tolerance [3]; however we did not observed drastic differences between genotypes on levels of these components. Photosynthetic pigments also did not show severe differences between genotypes [4]. The levels of anthocyanins, a stress induced pigment, under drought conditions were lower in tolerant genotypes suggesting a less stressed status in these accessions. We have observed that radicular system of some tolerant accessions (284 and 521) was larger, suggesting that root is directly involved in vetch drought tolerance (Additional file 3: Figure S1). These data suggest that, as expected, vetch have developed diverse adaptive strategies to cope with drought.

Based on our previous data that integrate phenological, yield components and harvest index data with the drought-associated parameters (SPAD index, stomatal conductance [13], we have selected genotypes 506 and 521 as representatives of sensitive and drought tolerant accessions, respectively for transcriptomic data.

### De novo assembly of transcriptomic data and functional annotation

To better understand genetic and molecular pathways involved in drought response mechanisms in the non-sequenced species *V. sativa*, we used high-throughput de novo RNA sequencing to assess the global changes of gene expression under drought conditions. In addition, to analyse potential drought tolerance adaptive mechanisms, we compared transcriptomic differences between drought-susceptible (506) and drought-tolerant (521) genotypes. An RNA sequencing analysis was performed to identify candidate genes involved in drought response and drought resistance. A pooled cDNA library from 12 mixed RNA samples was analysed with the Illumina HiSeq 2500 platform. From sequencing, a total of 660 million raw reads were obtained. After quality control and data clean-up process and de novo assembly, the resulting transcript sets were clustered (homology > 90%) into 63,878 high-quality unigene sequences; 5071 of which have > 1000 nt with an average length of 1015 nt and a N50 length of 605 nt. A summary of the Illumina-sequencing data, subsequent sequence assembly and length distributions are shown in Table 1, Additional file 3: Table S1 and Figure S2.

**Table 1:** Summary statistics of *Vicia sativa* transcriptome sequencing and assembly data

Total raw reads	660,973,924 (100%)
Mapped reads number (%)	566,017,832 (86.63%)
HQ reads (%)	118,855,982 (17.98%)
Properly paired reads (%)	118,855,982 (17.98%)
Splice reads (%)	4,496,190 (0.68%)
Total isotigs	63,878
max	5307
min	103
Total bases	31,709,744
Average	496
N50	605
N90	255
%GC	40.27
Number contigs > 1 kb	5071
Largest contig size	5307
Average lenght	1015

**Fig. 2:**
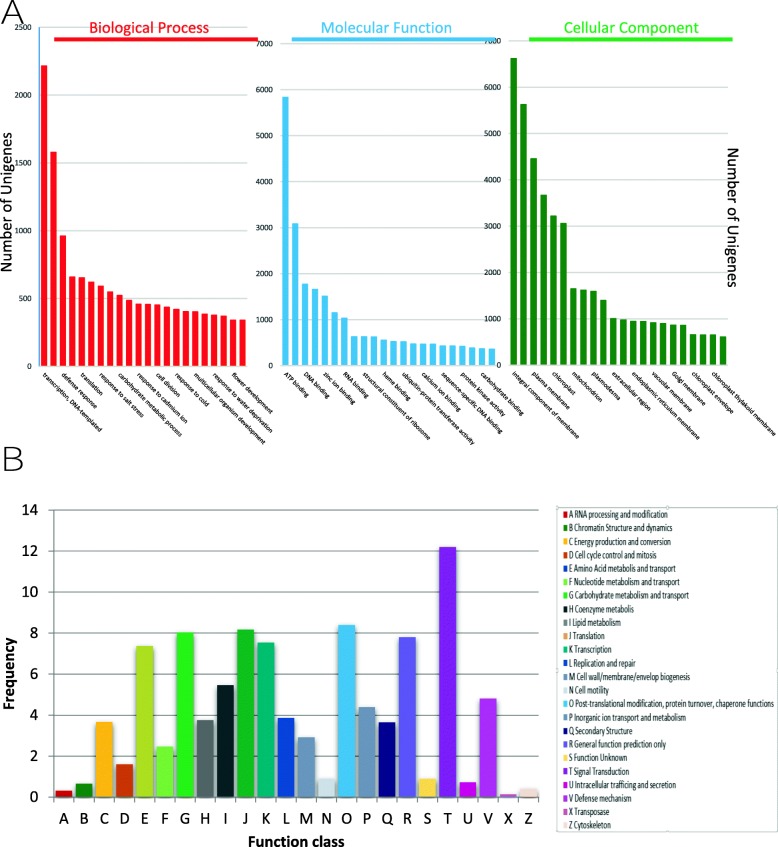
Major categories of GO and COG classification of the de novo annotated *V. sativa* transcripts. **a.** GO function classification of the annotated unigenes in *V. sativa*. The unigenes were summarized in major categories involved in Biological Process, Cellular Component and Molecular Function. **b.** Clusters of orthologous groups (COG) classification of the assembled common vetch unigenes. A total of 13,641 unigenes were classified into 24 functional categories according to their predicted gene products (COG cut-off E-value was 10^−5^)

After the sequence assembly, BLAST (BLASTN and BLASTX) searches were carried out against sequence databases (nt NCBI, RnaCentral and Uniprot) to infer gene functions using each unigene. Different unigenes were then annotated based on similarities to sequences available in these public databases. In total, 56,109 (87.8%) unigenes were successfully annotated in at least one databases, and 633 (1.1%) unigenes were annotated in all three (Table 2). For functional classification of the transcriptomes and annotation of the putative vetch gene function we used Gene Onthology (GO) assignment (Fig. 2a). A total of 29,691 (46.5%) unigenes were GO assigned to biological processes (BP), cellular components (CC) and molecular functions (MF). In the CC category, integral component of membrane (6616 unigenes, 10.4%), nucleus (5627 unigenes, 8.8%), and plasma membrane (4455 unigenes, 7.0%) were the largest GO subgroup. The most highly represented in MF subgroup were ATP binding (5835 unigenes, 9.1%), metal ion binding (3079 unigenes, 4.8%) and DNA binding (1768 unigenes, 2.8%). The subgroup BP was overrepresented by transcription, DNA-templated (2215 unigenes, 3.5%), regulation of transcription, (1578 unigenes, 2.5%) and defence response (960 unigenes, 1.5%).

**Table 2:** Summary statistics of *Vicia sativa* annotations in different databases

	Annotated	No hit	Total	%Annotated
Annotated in nt	55,615	8263	63,878	87.1
Annotated in rRNA_Central	1424	62,454	63,878	2.2
Annotated in Uniprot	30,388	33,490	63,878	47.6
Annotated in GO	29,691	34,187	63,878	46.5
Annotated in COG	11,662	52,216	63,878	18.3
Annotated in pfam	28,927	34,951	63,878	45.3
Annotated in KO	13,267	50,611	63,878	20.8
Annotated in at least one database	56,109	7769	63,878	87.8
GO annotations				
Cellular Component	62,091		187,721	33.1
Biological Process	55,906		187,721	29.8
Molecular Function	69,724		187,721	37.1

Clusters of Orthologous groups (COGs) function classifications of the assembled transcripts were also performed to predict the functions of annotated unigenes and gene products in Uniprot Database. Additionally, 13,641 unigenes were classified into 24 COG pathways (Fig. 2b). The most over represented were signal transduction (1664 unigenes, 12.2%), carbohydrate metabolism and transport (1096 unigenes, 8.0%) and general function (1064 unigenes, 7.8%). Other analyses were performed as Pfam Description and KEGG terms (KO) Pathway Enrichment that are shown in Additional file 1: Dataset S1.

### Functional analysis of differentially expressed genes (DEGs)

Gene quantification and differential gene expression were based on a negative binomial model. Analyses were carried out using the HTSeq-count 0.6.1p1 and DESeq2 methods, respectively [20].

**Fig. 3:**
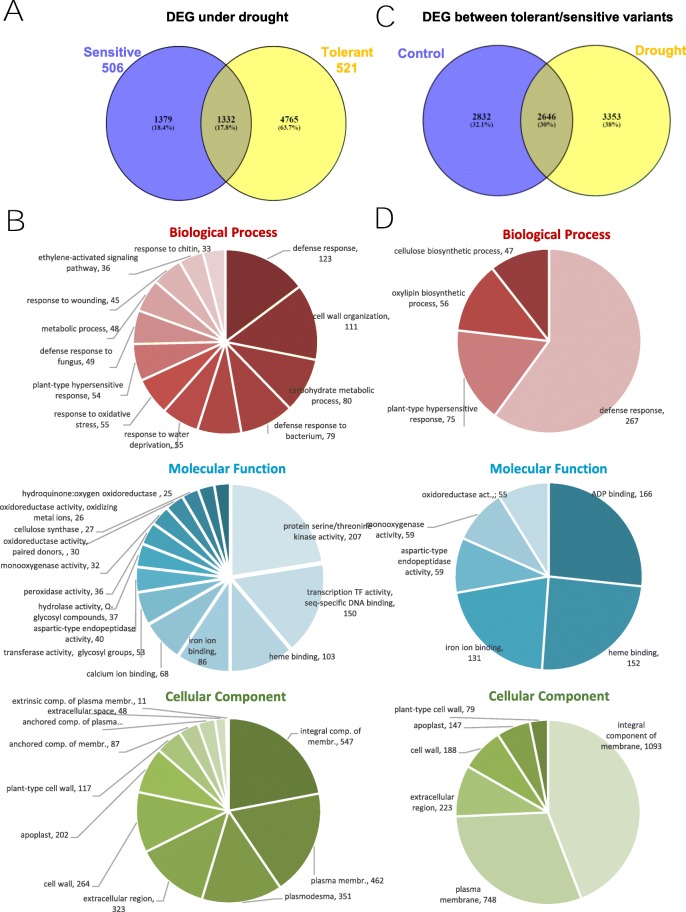
The common targets after drought treatments in tolerant and sensitive accessions and functional GO enrichment. **a.** The number in the Venn diagram (overlapping portion) represents the common targets deregulated under drought in sensitive 506 and tolerant 521 accessions. **b**. GO functional enrichment of the DEG (overlapping genes shown in (A) under drought between tolerant and sensitive plants in major categories involved in Biological Process, Cellular Component and Molecular Function in *Vicia sativa*. The numbers represent how many GO annotations are present in each different cellular processes. Results are based on the Blast2GO data mining. **c.** Venn diagram as in (A), but overlapping portion represents the common targets deregulated between tolerant and sensitive accessions under control or drought conditions. **d.** GO functional enrichment of the DEG (overlapping genes shown in (C), as indicated in B comparing overlapping DEGs between the accessions with different drought tolerance

Our transcriptomic data have allowed us to begin to understand the genetic mechanisms involved in drought response in common vetch. To evaluate gene response in drought conditions, we analysed the transcriptomic response in 4 week-old plants with or without watering in the different tolerant and sensitive candidates. A total of 2711 genes were differentially expressed between watered or drought treated plants 506, sensitive candidate accession, (|fold change| ≥ 2 and adjusted *p*-value (p-adj) FDR < 0.05), of which 1220 were upregulated and 1491 were downregulated. In the tolerant candidate 521, 6097 genes were differentially expressed. 3383 were upregulated and 2214 were downregulated after drought treatment. A total of 1332 transcripts were commonly deregulated in both accessions in these conditions (Fig. 3a). Moreover, the functional enrichment for each of the comparisons was calculated for the different categories of GO, KEGG (KO) Ontology, protein family collection database (Pfam) and Clusters of Orthologous Groups (COG) pathways. As expected, common DEG are enriched in GO categories involved in Biological Process as response to abiotic stresses including water deprivation, oxidative stress or abscisic acid ABA response, in addition to proline, cell wall and peroxide-related metabolism. The Cellular Component more overrepresented are plasma membrane and its integral components, plasmodesmata, extracellular region, apoplast and cell wall, that are components intrinsically linked to drought stress cellular response. Between the Molecular Functions with GO-enrichment, we found different redox and peroxidase activities, transcription factors and kinase activities (Fig. 3b). Same comparisons were done for others pathways. A more detail information of GO-enrichment for the different GO, KO, Pfam or COG pathways and statistical significance are shown in Additional file 2: Dataset S2. Briefly, common DEG are enriched in KO pathways of aquaporins TIP, peroxidases, chitinases, laccase, HSP70s, xyloglucosyl transferases or proline dehydrogenases and in Pfam *Leucine-rich repeat* (LRR) proteins, protein tyrosine kinases, Cu-oxidases, peroxidases, HSP70s, Cellulose synthases, extensins and others. Similar pathways are enriched when we analysed COG pathways (serine/threonine protein kinases, Leucine-rich repeat (LRR) protein, Cytochrome P450, Cu-oxidases, MIP, aquaporins and related.

To understand the adaptive response to drought stress in common vetch, we analysed the genes, and therefore the pathways, involved in the differential regulation between drought tolerant or sensitive accessions. A total of 5478 genes were differentially expressed between sensitive and tolerant candidate accessions in watered conditions and 5999 genes in drought conditions and 2646 DEGs in both conditions (Fig. 3c). Common DEGs are enriched in Biological Process GO directly involved in cellulose and oxylipin biosynthetic processes and plant-type hypersensitive and defence response. Plasma membrane and its integral components, extracellular region, apoplast and cell wall are the Cellular Component GO more overrepresented. Enriched-GO Molecular Functions are heme and iron ion binding, oxidoreductase and monooxygenase activity (Fig. 3d). Detailed functional enrichment of GO, KO, Pfam and COG pathways is shown in Additional file 2: Dataset S2.

Altogether, the comparative analyses of gene expression between irrigated and drought conditions in the two different accesions have allowed the identification of common drought response candidate genes. Furthermore, the analyses of the differential gene expression between tolerant and sensitive accessions both under irrigated (differential basal expression) and drought conditions (differential drought response) have allowed the identification of genes that potentially are responsible of adaptive mechanisms of drought tolerance.

### Differential expression of candidate genes to drought response: validation and expression analysis by qRT-PCR

To validate the reliability of microarray results, we select 14 genes (Table S2) which expression were analysed in tolerant and sensitive accessions in irrigated and drought conditions. VsGAPDH and VsUBC were selected as internal control genes, due to its stable expression. The comparison of the quantitative real-time PCR (RT-qPCR) data with that of the microarray showing a statistical correlation (r = 0.83), indicating that data are consistent (Additional file 3: Figure S3).

**Fig. 4:**
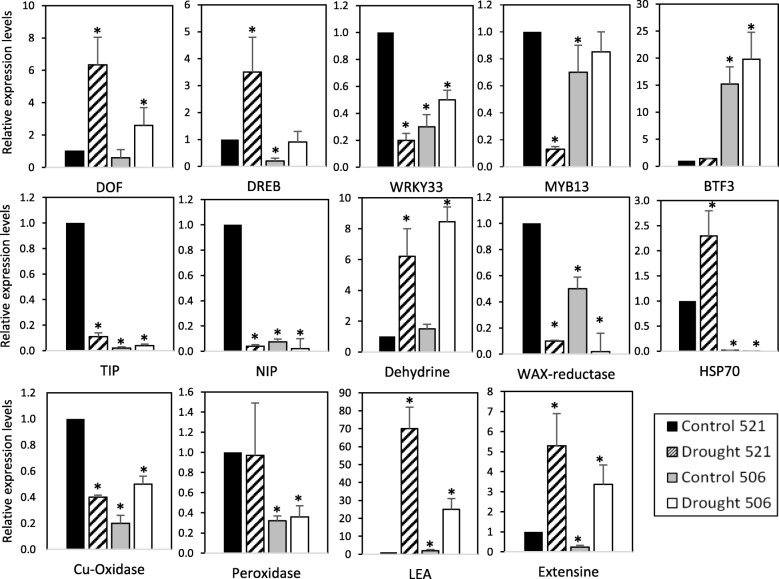
Gene expression of drought response genes. Expression levels of candidate genes for drought response and/or drought tolerance determined by real-time RT-PCR analysis using GADPH as standard gene for normalization in tolerant and sensitive *Vicia* greenhouse-grown 4-week-old plants under drought or control conditions. Values are the average (± standard deviation) of at least three assays. Asterisks show differences *p* < 0.05 (Student’s test) referred to control 521 tolerant plants

RNA-seq analysis shows that numerous genes show altered their expression, not only under drought but also between tolerant and sensitive genotypes. Among these DEGs, we have selected fourteen transcripts for qRT-PCR analysis (Fig. 4). We selected five TFs (VsDOF-like, DREB-like, WRKY33-like, Myb13, BTF3-NAC gene) identified to be involved in response to abiotic stress in other species [3, 21]. We also analysed the expression of aquaporins (NIP and TIP-type), the ABA response LEA-5 and dehydrins that have been reported to be involved in drought tolerance [22, 23] and proteins direct involved in redox homeostasis, as Peroxidase25 and Cu-oxidase-L-ascorbate oxidase. Our analysis also includes Extensin-1, chaperon HSP70 or WAX-biosynthesis fatty acid reductase (FAR), shown to be drought regulated in Arabidopsis [24].

As expected, the expression of vetch homologous of TFs DOF, DREB and BTF3 is induced under drought in both, tolerant and sensitive plants. However, important differences between both accessions were observed, also in untreated plants. Strikingly, TFs WRKY33 and myb13 are repressed in 521 accession and induced in 506 accession. Dehydrine, HSP70, LEA and Extensin-1 homologous are also induced under drought with differences between both accessions. Aquaporins TIP and NIP are repressed after drought treatment in 521 plants but remains at low expression level in 506 plants. Peroxidase25 expression has also differences between 506 and 521, plants but there is no induction under drought. Finally, Cu-oxidase-L-ascorbate oxidase is induced in sensitive accession but repressed in tolerant one, WAX-biosynthesis-FAR are repressed under drought in both plants, and HSP70 is induced under drought, but its expression is higher in tolerant plants (Fig. 4). These results suggest a complex network of the genetic response to drought, and point towards a complex differential regulation between the different genotypes.

Both aerial and radicular structures play an essential role in the response to drought, presenting different molecular and physiological mechanisms of action against this stress. For a more detailed study of the regulation of gene expression in these organs, we analysed the gene expression of the selected genes separately in root and aerial part. To extend the analysis to other genotypes previously described as tolerant or sensitive to drought, we included and analysed also the gene expression of these genotypes described in our work. We did not observe important differences in the DEG levels between the tolerant accessions 521 and 284, nor between the sensitive accessions 502 and 506, suggesting a correlation between gene expression and phenotypic differences of the drought-tolerant accessions (Additional file 3: Figure S4). However, specific tissue-associated expression behaviour has been observed in some genes. LEA induction after drought is more relevant in aerial than in radicular part. Aquaporin TIP is repressed in aerial part, but is induced in root. HSP70 induction after drought in tolerant accessions is more relevant in root than in aerial part. Expression of Peroxidade25 remains constant in aerial part and differences are only observed in root. These differences are partly explained by the expression of these genes on a tissue specific way and by the action of different regulatory mechanisms of gene expression in the analysed organs. As expected, a complex scenario has been opened to explain the differences in the genetic expression in different tissues and between accessions with different drought tolerance, suggesting an intricate and multifaceted network of the genetic response to drought.

### Identification of potential drought-response gene variants: SNPs and SSRs

RNA-seq has proven to be an accurate, reproducible and high-throughput method to identify genetic variants such as SNPs and SSRs, especially in non-model species for which genomic resources are unavailable. This strategy has been useful, allowing the predictive mapping of QTLs associated to traits of interest and the use of marker-assisted selection (MAS) strategies [25].

**Fig. 5:**
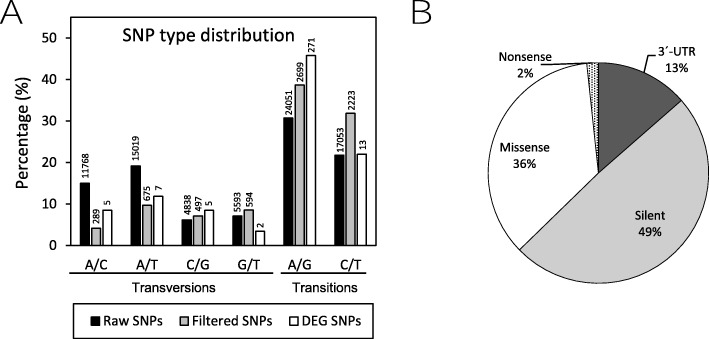
Overview of the identified Single Nucleotide Polymorphism (SNPs). **a.** Distribution of putative SNPs in *V. sativa* transcriptomes by type of transversion/transition. Numbers at top of bars indicate total number of SNPs. **b.** The pie chart showing functional categories of the detected SNPs present in DEGs, including synonymous mutations (silent), nonsynonymous mutations (nonsense and missense) and changes affecting 3′-UTR

The transcriptome sequences were mined for SNPs markers, using HaplotypeCaller. In a first approach, 78,322 SNPs in 32,874 unigenes were detected. To obtain high confidence results in the subsequent analyses, stringent criteria were applied for SNP filtering selecting for loci present in both genotypes and genotyped in at least 60% of the individuals in each population, excluding SNPs with a minor allele frequency (MAF) < 0.05 within two populations or polymorphic loci with more than two alleles that could be artefacts or sequencing errors. After this filtering, 7246 high-quality SNPs in 4230 unigenes were obtained. Therefore, about 6.6% of total vetch transcripts contained putative SSR sequences. The transversions and transitions frequencies (Fig. 5a) were similar to those observed in other plant species [26]. Identification of SNPs located in CDSs is essential for association with relevant agronomic traits of economic interest. To establish a correlation between differential gene expression between the tolerant and drought sensitive accession and the presence of potential functional markers, we analysed the presence of SNPs in DEGs with criteria of high stringency. A total of 59 SNPs (67.8% transitions/32.2% transversions) were found in 36 differentially expressed transcripts with significant statistical significance. Of these 59 SNPs, 8 changes were found in 3′-UTR regions and 51 changes in a predicted open reading frame (29 silent mutations; 21 missense mutation and one nonsense mutation). Detail information of specific data and frequencies are indicated in Table 3 and Fig. 5b). The percentage of nonsynonymous SNPs in coding regions is comparable to ratios found in other eukaryotic studies (review in [27]). Significantly, some of these nonsynonymous SNPs are into transcripts with potential homology with genes involved in abiotic stress response, maintenance of redox and osmotic homeostasis, root development photosynthesis and important regulatory proteins as transcription factors and signalling kinases (Table 3).

**Table 3:** Identification of SNP candidates in DEG genes, indicating position and change into de transcript

ID Transcript	ID SNP1	Position	Change	ID SNP2	Position	Change	ID SNP3	Position	Change	*p*-value	Description
Transcript_0322	SNP_0118	375	A > G (3’U)	SNP_0119	555	T > A (S)	SNP_0120	590	T > C (MS)	8.76807E-06	Bax inhibitor-1 family protein
Transcript_0328	SNP_0121	115	T > A (3’U)	SNP_0124	391	G > A (S)				1.25871E-08	Ribosomal protein L27
Transcript_0419	SNP_0156	559	A > G (S)	SNP_0157	586	T > C (MS)				1.14691E-06	Photosystem II reaction Center PSB29
Transcript_0702	SNP_0261	791	A > G (3’U)							8.64592E-06	alpha/beta-Hydrolases superfamily protein
Transcript_0989	SNP_0352	175	A > T (S)							8.9403E-06	Cu + −exporting ATPase
Transcript_4678	SNP_1069	413	A > G (S)	SNP_1070	332	C > G (S)				2.87933E-07	copper-transporting ATPase RAN1 isoform X2
Transcript_6171	SNP_1231	368	C > T (S)	SNP_1232	418	T > A (MS)	SNP_1233	540	C > G (MS)	2.31921E-06	E3 ubiquitin-protein ligase TRIP12
Transcript_8874	SNP_1459	717	C > T (S)	SNP_1460	520	T > C (MS)				1.48291E-06	Pleckstrin homology (PH) domain superfamily protein
Transcript_9613	SNP_1480	590	G > C(MS)	SNP_1481	674	T > C(MS)	SNP_1482	702	A > C(NS)	2.15232E-08	Ribosomal L38e protein family
Transcript_10054	SNP_1541	769	T > A(3’U)	SNP_1542	188	C > T(3’U)				6.32482E-06	26S protease regulatory subunit 6B
Transcript_10860	SNP_1609	242	A > G(MS)							3.71096E-06	Pentatricopeptide repeat (PPR) superfamily protein
Transcript_11543	SNP_1624	161	C > T(MS)							6.05323E-07	fructose-bisphosphate aldolase 1
Transcript_11804	SNP_1729	989	A > G (S)	SNP_1730	195	G > A(S)				7.03222E-06	Actin-11 related
Transcript_15438	SNP_2477	626	A > G(S)							1.51852E-06	pleckstrin homologue 1
Transcript_20917	SNP_3006	716	T > C (MS)	SNP_3007	725	T > A(MS)				8.86323E-07	Lipase_3
Transcript_20941	SNP_3015	250	T > G (MS)	SNP_3016	704	G > A (S)				2.19552E-06	GTP-binding protein involved in stress response
Transcript_21213	SNP_3100	169	C > G (S)							6.76584E-06	Signal recognition particle, alpha subunit
Transcript_21241	SNP_3115	481	C > A (3’U)	SNP_3116	484	A > G (S)				1.36926E-06	manganese superoxide dismutase 1
Transcript_21548	SNP_3225	411	T > C(S)							6.94317E-06	DNA-binding enhancer protein-like protein
Transcript_21929	SNP_3368	352	A > T(S)							2.96764E-06	p450
Transcript_29618	SNP_4470	85	A > G(MS)							5.26296E-06	mannosyl-oligosaccharide 1,2-alpha-mannosidase MNS1
Transcript_43164	SNP_5625	528	C > T(S)							7.99581E-07	SOUL heme-binding family protein [
Transcript_43425	SNP_5679	292	C > T(3’U)	SNP_5680	297	A > G(3’U)				5.15776E-08	ATPase, V1 complex, subunit B protein
Transcript_44989	SNP_5866	524	T > G (MS)							3.94602E-06	ATP-dependent Clp protease ATP-binding subunit
Transcript_48019	SNP_5992	338	A > G(S)							2.25642E-06	Transcription Factor HAT9; Homeobox-leucine zipper protein
Transcript_50340	SNP_6030	56	G > A(S)							8.21592E-06	photosystem I subunit XI
Transcript_50427	SNP_6051	697	A > G(S)							3.34132E-07	Lipase_3
Transcript_50487	SNP_6054	292	A > G(S)	SNP_6055	322	G > A(S)	SNP_6056	364	G > A(MS)	3.6413E-06	Glycolytic
Transcript_50526	SNP_6062	285	G > A(MS)	SNP_6063	318	G > A(MS)				9.15844E-06	Acyl transferase/lysophospholipase superfamily protein
Transcript_50907	SNP_6132	245	A > G(S)	SNP_6133	254	G > A(S)	SNP_6134	317	G > A(S)	5.88456E-06	ADP/ATP carrier 2
Transcript_51136	SNP_6181	431	C > G(MS)							8.48917E-06	membrane-associated progesterone receptor component
Transcript_55265	SNP_6483	519	A > G(S)	SNP_6484	553	G > A(MS)				7.86331E-06	Ribosomal_L27
Transcript_55491	SNP_6528	363	C > A(S)							1.04189E-06	enolase
Transcript_55845	SNP_6592	293	C > T(MS)							2.53713E-06	Yellow Stripe like 6
Transcript_55919	SNP_6606	744	A > C(S)	SNP_6607	747	A > C(S)				3.37812E-07	Annexin;response to water deprivation,
Transcript_61815	SNP_7139	371	G > A(S)							8.63893E-08	lipoxygenase

Note: Parenthesis in Change Column indicates 3′-UTR regions (3’U), silent mutations (S), missense mutation (MS) and nonsense mutation (NS). Note the presence of more than one SNPs in some transcripts

**Fig. 6:**
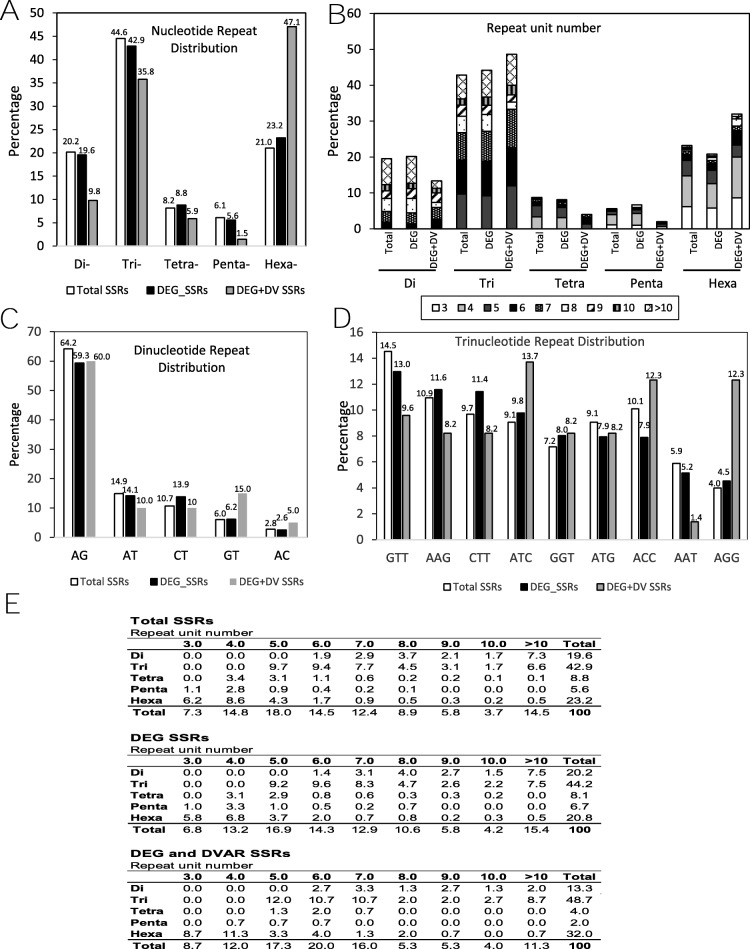
Overview of the identified Simple Sequence Repeats (SSRs). **a.** Distribution of putative SSRs by length of unit repeat, excluding mononucleotide repeats. **b.** Distribution of putative SSRs Repeat unit number in the Total SNPs, SNPs present in Differential Expressed transcript (DEG) and SNPS present in DEG and differentially present between accessions (DEG + DV). **c-d.** Distribution of the main sequence of dinucleotide or trinucleotide repeats, respectively. **e.** Summary information on frequencies of different SSR repeat motif types related to variation of repeat unit numbers in *V. sativa* SSRs, excluding mononucleotide repeats

SSRs are molecular markers of great application in processes such as MAS, varietal identification and genetic mapping. Using HipSTR algorithm, all the 63,878 transcript from the genotypes of the two accessions were searched for the presence of SSR. A total of 6848 SSRs in 5642 (8.8%) transcripts were identified. Identified SSRs present motif lengths ranging from one to six bp. Data analysis of the SSR motifs revealed that most abundant repeats were trinucleotide (32.3%) and mononucleotide (24.5%). Di-, tetra-, penta-, and hexanucleotide repeats are 14.4, 6.1, 4.1 and 18.2% of the total number of SSRs, respectively. These frequencies, excluding mononucleotide repeats, are shown in Fig. 6a and b. These data are similar to those obtained by analysing other legume species [25, 28]. Frequencies of the most abundant dinucleotide and trinucleotide motives were analysed (Fig. 6c and d). Similar distribution are observed in the total identified SSRs, the SSRs in differential expressed transcripts (DEG-SSRs) and the SSRs in differential expressed transcripts and differential present between accessions or varieties (DEG-DV-SSRs; Fig. 6c, d). The complete data distribution of motives and unit number are summarised in Fig. 6e.

The comparative analysis of the 54 SSRs, found in 52 different transcripts with polymorphic variants in the different accessions (DEG-DV-SSRs), and that present a differential genetic expression among them, shows us that they are present in transcripts that encode regulatory proteins, such as signalling phosphatases or kinases, TFs involved in abiotic stress (WRKY, MAC, myb and bHLH family members), hormone response genes, chaperons, and proteins directly related with photosynthesis and redox homeostasis (Table 4).

**Table 4:** Identification of SSRs candidates in DEG genes, indicating position and repeat motif into de transcript. Note the presence of more than one SSRs in some transcripts

ID Transcript	ID SSR	Position	Motif	Description
Transcript_00199	SSR_1396	826	GTT	zinc finger CCCH domain-protein 29/Salt stress
Transcript_01630	SSR_943	301	ATC	aspartyl protease family protein
Transcript_02098	SSR_1543	520	ACC	AAA-ATPase
Transcript_02315	SSR_1808	938	AACATT	Unknown chloroplastic
Transcript_02904	SSR_2429	76	AAGGAG	Unknown
Transcript_03098	SSR_2626	360	ATG	WRKY57
Transcript_03641	SSR_3317	324	ATTCCC	U-box domain-containing protein 4-like
Transcript_04246	SSR_4169	248	ATCTTC	BIG GRAIN 1-like B
Transcript_05713	SSR_5677	706	CGGGCT	Unknown
Transcript_05713	SSR_5678	822	ATGTTG	Unknown
Transcript_06440	SSR_6348	141	AC	Unknown
Transcript_08212	SSR_6623	195	AAAAC	Unknown
Transcript_10045	SSR_29	164	CTG	RNApol II-adapter
Transcript_10292	SSR_81	613	CT	BTB/POZ domain protein Ubiquitination
Transcript_10364	SSR_92	126	AAT	Unknown
Transcript_10647	SSR_141	295	GT	Unknown
Transcript_11503	SSR_275	755	ATC	S/T-protein phosphatase 6 regulatory subunit
Transcript_11857	SSR_355	720	ATG	BEL1-related homeotic protein
Transcript_12126	SSR_408	174	GTT	homeobox-leucine zipper protein ATHB-6-like
Transcript_12316	SSR_439	106	AGTGGT	ethylene-responsive transcription factor ERF106
Transcript_15579	SSR_851	407	AGG	geranylgeranyl diphosphate reductase
Transcript_17222	SSR_1068	1548	CTT	Unknown
Transcript_17403	SSR_1094	574	ATCGTC	histone H2A.Z-specific chaperone CHZ1-like
Transcript_19735	SSR_1368	147	AAC	DnaJ-class molecular chaperone withZn finger domain
Transcript_19853	SSR_1389	230	ATC	GTP-binding protein OBGC, chloroplastic
Transcript_19875	SSR_1393	260	CT	S/T-protein kinase PBL11
Transcript_21213	SSR_1592	378	AATGGT	Unknown
Transcript_22868	SSR_1784	740	GGTGTT	PP2C
Transcript_23073	SSR_1802	261	ACAT	6P-fructokinase
Transcript_24458	SSR_1944	276	ACC	Unknown
Transcript_28981	SSR_2422	798	GGGTTT	P450-dependent fatty acid hydroxylase
Transcript_29846	SSR_2497	184	CTT	TOM1-like (Target Of Myb1 Like 1 Membrane Trafficking Protein)
Transcript_30513	SSR_2582	379	AAG	Unknown
Transcript_30774	SSR_2613	116	AAG	Unknown
Transcript_31019	SSR_2634	672	CGT	DNA topoisomerase
Transcript_34830	SSR_3117	145	ATC	PPR|PPR_3
Transcript_34933	SSR_3128	258	AGCAGG	NAC53
Transcript_35562	SSR_3201	69	AAGATG	RNA pol II
Transcript_35581	SSR_3202	143	GTT	E3 ubiquitin-protein ligase BOI
Transcript_37552	SSR_3464	397	CTT	bHLH13 TF
Transcript_37747	SSR_3489	237	AGG	URO-D (Chlorophyll biosynthesis9
Transcript_37747	SSR_3490	394	ATG	URO-D (Chlorophyll biosynthesis9
Transcript_40868	SSR_3958	354	ACC	Serine/threonine protein kinase
Transcript_43126	SSR_4267	1028	AGG	fasciclin-like arabinogalactan protein 8-like
Transcript_45977	SSR_4565	158	AT	glucan endo-1,3-beta-glucosidase 4
Transcript_46871	SSR_4655	313	AGGTGG	Methyltransf_29
Transcript_48008	SSR_4739	204	ATG	pyruvate dehydrogenase E2 component
Transcript_53459	SSR_5294	262	GGTTGT	stress-associated protein 5 zinc finger A20 and AN1 domain-containing
Transcript_56560	SSR_5623	430	CCTGGG	Unknown
Transcript_57761	SSR_5745	298	ACC	protein ALP1-like
Transcript_58453	SSR_5821	713	AGG	LRR receptor-like serine/threonine-protein kinase
Transcript_59358	SSR_5906	671	AG	tyrosine-protein phosphatase
Transcript_60192	SSR_5992	115	AGT	Oxidoreductase
Transcript_62649	SSR_6230	182	AGTTGG	Unknown

Both SNPs and SSR markers present in coding sequences of genes which are differentially expressed in tolerant and sensitive accessions under drought we are generated in this analysis can facilitate marker-assisted selection for vetch improvement programs, because these may be associated with functionally genes, are cost-effective, and are easily transferable to related species, for its conservation. Future analysis should be done to characterise the potential value of these markers in future strategies for prediction of relevant traits as drought, or accelerate breeding programs searching for drought tolerant accessions.

To validate the SSR/SNPs identified in this study, ten primer pairs were designed to test the amplification of fragments containing the putative SNPs and ten to validate SSRs (Table S3 y S4, respectively). Seven out the 10 SNPs and seven out the 10 SSR provides a single amplicon of the expected fragment size (Additional file 3: Figure S5). Future approach could validate the functionality of these molecular markers as drought tolerance associated markers.

## Discussion

Growing global population and climate change involve multiple challenges on crop improvement. In this scenario, a better understanding of drought response mechanisms and associated traits is essential for efficient crop growth mitigating water-limited conditions. Previous studies reaffirmed that drought tolerance is a trait of high complexity under the control of many genes. In this context, the understanding of how plants respond to drought stress at the molecular level is essential for developing improved genotypes which would perform well under water-limited conditions. Exploring the biodiversity present in crop genetic resources is an essential tool to address the identification of drought tolerant accessions. Germplasm banks collect and maintain genetic diversity in collections that contain local accessions or landraces, commercial cultivars, hybrids and related wild species. Nevertheless, the main limitation of using these collections is the lack of characterisation data, essential for analysing their genetic diversity, identifying potential valuable traits and selecting local accessions for breeding or for farmer direct use. With the development of novel genomic approaches, genotyping is becoming efficient and cheap. NGS techniques have allowed the large-scale screening to identify ambient relevant novel genes and genetic pathways [29]. The development of markers from coding regions may allow the tagging of QTLs for relevant agronomical important traits [30]. Despite its ecological and economic relevance, there is an important lack of genomic resources for *V. sativa*, limiting the advancement in the improvement in this crop and the characterisation of collections at molecular level. Hence, the generation of high quality genomic information is essential to understand the molecular mechanisms underlying desirable agronomic traits. In addition, a better integration of genotypic and phenotypic data will be useful for the development of genomic tools for trait prediction over vetch collections. In addition, the knowledge of genetic bases of ambient adaptation is essential for a rational conservation and utilization of plant genetic resources under climate change conditions [31].

In this work we have developed de novo assembly and gene annotation of transcriptome from two vetch genotypes: drought-tolerant and drought-sensitive and their drought response. Our results have helped to understand, not only the molecular mechanism associated to drought but also the morphological, physiological and biochemical changes associated with this stress.

The drought-tolerant accessions have greater stomatal conductance than the sensitive ones. Tolerant accessions present lower rate of evapotranspiration and greater ROS production, especially in drought conditions. These data suggest that vetch stress tolerance could be due to reduced transpiration rate mediated by alteration of stomatal closure and stomatal ROS production dynamics.

Detailed understanding of plant molecular responses to environment stress is crucial to make stress tolerant crops. Two main groups of drought inducible genes are been identified as general regulators [32]. First group comprise proteins mostly involved in stress tolerance, including antifreeze proteins, enzymes for osmolyte biosynthesis, key water channel proteins, sugar and proline transporters, detoxification enzymes, chaperones and late embryogenesis abundant (LEA) proteins. The second group includes regulatory proteins, as transcription factors, protein kinases and phosphatases, and other signalling molecules. Transcription factors (TF) play an essential role in controlling gene expression in drought signalling pathways, as they can regulate expression of several genes in an efficient and rapid manner and may constitute complex gene networks [32]. Most of these drought response pathways have been characterised in Arabidopsis. However, the main regulatory mechanisms are conserved in other crop plants. Our transcriptomic analysis shows conserved drought response mechanisms in vetch. Numerous signalling serine/threonine kinases, phosphatases and members of TF families involve in drought response are also deregulated in common vetch under drought. We also demonstrate that the drought response of some genes (DOF, DREB, WRKY33, MYB13 and BTF3) is regulated in a tissue-specific manner. And the most important, these genes present a differential regulation between drought-tolerant accessions and sensitive ones, suggesting an important role in adaptation or tolerance to drought conditions. Similar behaviour is also observed in the expression of some vetch aquaporins, dehydrines and other LEA proteins, especially in root. Expression of these genes was shown to be regulated in several species in response to drought or ABA [22, 23]. Taken together, these results suggest a complex network of the genetic response to drought in common vetch*,* pointing towards a differential regulation between the different genotypes on a tissue specific manner by the action of different regulatory mechanisms of gene expression. During the preparation of this manuscript, a transcriptomic work on common vetch after an in vitro polyethylene glycol (PEG) treatment has been published. PEG treatments partially mimic the effect of drought. Although the conditions in which these treatments have been performed are not comparable to those presented in our work, some genes and activated routes are similar to those identified in our studies. In both analyses there is a transcriptional enrichment of relevant GO Biological Process categories as “oxidoreductase activity”, “redox process”, “metabolic process” “cell wall modification” or “carbohydrate metabolic process” [33].

Crop selection by conventional methods has been traditionally used to increase stress tolerance and yield, however, drought tolerance selection based on phenotype analysis is complex and is highly influenced by environmental changes. The integration of our functional and genetic information contributes to the development of genomic tools for drought tolerance prediction, allowing their potential used in future strategies to accelerate breeding programs and in the development of predictive genotypic and phenotypic analysis for further use in gene-banks. Similar predictive strategies have been successfully used in other legumes [34–36]. Further analyses have to be done to validate the potential use of the identified polymorphic variants of this work as predictive tools in drought prediction.

## Conclusions

Our studies may help to understand the genes and molecular mechanisms associated with drought stress in common vetch. In addition, we identify over 100 potential drought associated polymorphism, as SNPs or SSRs, which are differently present in drought and tolerant genotypes. The analysis of these polymorphic variants, as molecular markers for trait prediction, would enable the development of genomic tools for future engineering strategies by screening of germplasm crop collections for traits related with crop drought resilience, adaptability or yield in vetch. In addition, the genomic characterisation of gene-bank collections will increase their value in farming and breeding sectors in the light of different environmental contexts, especially in the context of adaptability to changing climate.

## Methods

### Plant materials

The original source of plant material (*Vicia sativa* seeds) used in this study is the Spanish National Plant Genetic Resources Center (CRF) belonging to the National Institute for Agricultural and Food Research and Technology (INIA). This material is deposited in the publicly available CRF-INIA seed bank. All the data about formal identification of the plant material, original source, passport data and the accesion numbers are available at http://webx.inia.es/web_inventario_nacional/Introduccioneng.asp. Over field grown common vetch accesions of this core collection we carried out an analysis of parameters associated to drought-response including chlorophyll content, leaf colour, cover temperature, epicuticular wax content, residual respiration and specific weight [16]. These data have allowed us the selection of candidates with good response to drought conditions. Upon the field screening data, genotypes number 284, 510 and 521 were identified as drought tolerant accessions, and genotypes number 502, 506 and 545 (Verdor, commercial variety) were identified as drought sensitive ones. Deposition code numbers of the accesions are: BGE037817; BGE005449; BGE004375; BGE014897; BGE022207 and Verdor. The correspondences between the code used in this work, at the CRF collection and at National Inventory: 284/NC081023-BGE037817; 502/NC010040-BGE005449; 506/NC013296-BGE004375; 510/NC018857-BGE014897; 521/NC043873-BGE022207 and 545-Verdor commercial variety.

### Field grown conditions and measures of stomatal conductance

In this work common vetch was grown in field only for seed amplification and for stomatal conductance measures. Plants were sowing for a field analyses in “Finca La Canaleja”, Madrid (602 m a.s.l.; 40°30′54″N/03°18′42″W). The meteorological conditions of this region involve an average annual rainfall of 420 mm and an average daily temperature of 13.7 °C. The soil characteristics of this field: Calcium alfisol, loam, moderately alkaline (pH 8.4), organic carbon (0.6%), saturation of bases (100%). The average rainfall from October-2016 to June 2017 was 203 mm and 374.4 mm in the same period of 2018. For these assays no wild samples were collected and field studies was conducted in accordance with local, institutional, national, and international legislation.

Plants were sowed in November and stomatal behaviour was measured in the field when 50% of plants were flowering on a total of 10 plants per accession and in five leaves per plant on the first expanded leaf on sunny days, without clouds or wind, from 10 am to 12 pm in April 2017 and four weeks later, in May 2017, with the 50% of plants are fully formed pod. These measurements were carried out in the same way in April–May 2018. Stomatal conductance (mmol m^− 2^ s^− 1^) was done in duplicate and repeatedly during consecutive days using a steady-state leaf porometer (SC-1, Decagon-Devices, LabFerrer, Spain).

### Greenhouse grown conditions

Analysis of stomatal aperture, stomatal index, ROS measurements, water loss rate determination, transcriptomic assays and analysis of pigments, osmolytes and epicuticular waxes were done with plants grown in greenhouse conditions. Vetch plants were grown in greenhouse under a 16 h light/8 h dark photoperiod at 22 ± 1 °C and plants were grown in pots (15 cm diameter) with soil.

For drought treatments, one week-old plants were watered and subsequently greenhouse-grown for three additional weeks without additional watering. The assays were repeated at least four times.

### Stomatal aperture bioassays, stomatal index and ROS measurements

Stomatal measures were developed as previously described by Del Pozo and Ramirez-Parra [37]. Leaves from 4 greenhouse-grown week-old plants were incubated in buffer containing 50 μM CaCl_2_, 10 mM KCl and 10 mM MES/KOH (pH 6.10) buffer. To induce stomatal opening, the strips of abaxial epidermis from leaves were incubated in the light during 3 h. At least 100 cells from five different leaves were photographed in an Axioskop2 plus microscope (Zeiss), and processed with the ImageJ NIH software. For stomatal index and cell density calculation, at least 500 total cells were analysed. ROS production was detected using carboxifluorescein diacetate (CFDA) essentially as previously described by Miao et al. [38]. The epidermal tissues were incubated in 50 mM Tris-ClH (pH 7.2) buffer containing 10 μM CFDA in the dark for 10 min. Detection was done in an Axioskop2 plus epifluorescence microscope (Zeiss) and analyzed using Quantity One (BioRad).

### Water loss rate determination

Plant water loss rate was measured using a minimum of 15 greenhouse-grown units of each Vicia accession and were analyses as described by Del Pozo and Ramirez-Parra [37]. Detached aerial part of plant of 28 day-old were immediately weighed (FW, fresh weight). To estimate the dessicated weight (DesW) plants were placed at laboratory conditions (22 °C and relative humidity 45%) and weighed at indicated times. Plants were oven-dried (65 °C) during 2 days to achieve a constant dry weight (DryW). Percentage of leaf WC (Water content) was calculated as 100 × (DesW−DryW)/(FW − DryW).

### Pigments, osmolytes and epicuticular wax determination

For the determination of these components, we used 4-week-old leaves from greenhouse-grown plants. Chlorophyll content was determined spectrophotometrically after acetone extraction as described by Arnon [38]. Anthocyanins were extracted from plants using the acidified methanol method of Wade et al. [39]. Osmolyte levels were quantified as previously described [37]. Briefly, total soluble sugars were determined by using the anthrone reagent, as described by Yemm and Willis [40]. Free proline levels were quantified following the method described in Bates et al. [41]. Epicuticular wax content was determined using glass vials for the assay as described by Ebercon et al. [42].

### RNA extraction and library construction for transcriptome analysis

Root and aerial part were collected from 4 different 4 week-old greenhouse-grown plants (watered control or drought-treated as previously described). Assays were done in triplicate. Equal weights of material were pooled prior to RNA extraction. Total RNA was extracted using Trizol (Invitrogene) and Plant RNA Extraction Kit (Omega). The quality and quantity of the RNA has been determined in Bioanalyzer 2100 and Qubit 3.0. For libraries preparation the Poly(A) + mRNA fraction was isolated from 10 μg of total RNA and cDNA libraries were obtained following Illumina’s recommendations. The quality of the libraries was analysed in TapeStation 4200, High Sensitivity assay; the quantity of the libraries was determined by real-time PCR in LightCycler 480 (Roche). The pool of the 12 libraries was sequenced by paired-end sequencing (100 × 2) in Illumina HiSeq 2500 sequencer (3 flow-cells) using Sistemas Genomicos Sequencing Facilities (Valencia, Spain).

### Data analysis for transcriptome sequencing: de novo assembly and functional annotation

The quality of the raw data was checked using FASTQC tools [43]. Trimmer and preprocessing steps were applied using FastqMcf [44] and *in-house* scripts. Sequences were assembled with Oases [45] with different K-mer sizes. The best assemblies from Oases were merged with Cap3 [46]. The best assemblies were chosen by the best N50 [47].

For functional annotation, transcript sequences were annotated with BLASTN and BLASTX [48] against non-redundant nucleotide sequence (Nt) from National Center for Biotechnology Information (NCBI), BioSystems database [49] and RnaCentral [50], and against Uniprot [51], with an cut-off E-value of 0.001 and *in-house* scripts to clean the sequences with poor homology. For functional assignment we used the following publicly available protein databases: Protein family (Pfam), Gene Ontology (GO), Eukaryotic Orthologous Groups of proteins (KOG), and the Kyoto Encyclopedia of Genes and Genomes (KEGG). Results of sequencing, transcriptome assembly, gene prediction and annotation of 12 libraries merged as only one. Finally, reads obtained from the sequencing of different samples and treatments were mapped and compared to the newly created de novo assembled library using Tophat2 v2.1.0 [52], cleaning of low quality reads was done with Samtools [53] and Picard Tools (http://picard.sourceforge.net). Gene quantification and differential gene expression were carried out using the HTSeq-count 0.6.1p1 and DESeq2 methods, respectively [20].

### Differential expression analysis and functional enrichment

To corroborate different biological replicates, distances and correlation of data from different samples were analysing considering the complete transcriptome normalized with Principal Component Analysis with the statistical software R [54]. Differential expression studies between different samples were analysed with Phython and R based statistical software. Differential expression analysis was performed with the DESeq2 algorithm [54], using a negative binomial distribution for determination of statistically significance. In this analysis, the isoforms with |Fold Change| ≥ 2 and a threshold FDR with corrected *P*-value of less than 0.05 were assigned as differentially expressed. For functional enrichment analysis (GO and KEGG) of DEGs, hyper-geometric test using data from Uniprot and COG was applied within the obtained blastx results. Threshold was set as FDR with corrected P-value < 0.05 to determine a functional category as statistically significant.

### Quantitative real-time PCR analysis

RNA samples extracted as indicated for transcriptomic analysis (4 week-old greenhouse-grown plants) was used for cDNA synthesis. Retrotranscription was carried out with the High Retrotranscriptase Kit (Biotools). The Applied Biosystems ABI 7300 System with the FastStart DNA Master SYBR Green I (Roche) was used for real-time quantitative RT-PCR. The concentration of GlycerAldehyde-3-Phosphate-DeHydrogenase (Vs*GADPDH)* and Ubiquitine (*VsUBC*) gene levels were used for normalization, due to the relatively stable expression in transcription profiles and empirically control data. Data derived from two independent experiments were carried out in triplicate. Sequence primers are specified in Table S2.

### Identification of new variants

For variant analysis and SNPs identification, we used GATK HaplotypeCaller v4.0.2.1 applying default-setting criteria of best practices from Genome Analysis Toolkit (GATK) Variant Discovery in High-Throughput Sequencing Data. MatrixEQTL was used for test association between genotype and gene expression using linear regression with either additive or ANOVA genotype effects.

For identification of putative SSRs from assembled transcripts and differentially expressed transcripts, we used algorithm HipSTR a novel haplotype-based method for robustly genotyping and phasing STRs from Illumina sequencing data (https://hipstr-tool.github.io/HipSTR) The sequences were searched for perfect mono-, di-, tri-, tetra-, penta- and hexa-nucleotide motifs with a minimum of three repeats.

### Statistical analyses

Statistical analyses of the data were performed using Excel add-in Real Statistic pack. One-way or two-way ANOVA with Tukey-HSD test was used for testing differences between multiple samples (*p* < 0.05).

## Supplementary information

**Additional file 1: Dataset S1.** Excel tables with *V. sativa* Uniprot annotation and functional classification assignment of the assembled unigenes and in GO, KEGG, Pfam and GOG terms. https://drive.google.com/open?id=153IX8pv-CxL6G4SDoKjP17WQ26FMeY9x

**Additional file 2 Dataset S2.** Excel tables containing information of DEG in different experimental conditions as indicated (drought versus control, and tolerant versus sensitive variants in both conditions) and statistical analysis of functional enrichment in categories: GO, KEGG, Pfam and GOG terms. https://drive.google.com/open?id=1WnnFJTsmCVBGFvevDgQCOki7z0dz1e8l

**Additional file 3 Figure S1.** Evaluation of epicuticular wax content, root and aerial weight, osmolyte levels and pigment content for accession identified as drought tolerant (284, 510 and 521 identified with plain colours on the histograms bars of all the figures) and drought sensitives (502, 506 and 545 identified with ornamented wefts on the histogram bars of all the figures). **A.** Epicuticular wax content from 4-week-old leaves from greenhouse-grown plants. **B.** Weight of aerial and radicular part of different 4-week-old greenhouse-grown varieties. **C-D.** Soluble sugars (lower panel) and free proline (upper panel) content were determined on 4-week-old greenhouse-grown plants under control condition or drought treated (3 experiments; *n* = 10 plants/each). **E-F.** Pigment content: Anthocyanin levels (E) or chlorophyll a and b levels (F) from 4 week-old greenhouse-grown plants under control condition or drought treated (3 experiments; *n* = 20 plants/each). **A-E.** Values are means ± sd. Different letters indicate significantly differences analysed by ANOVA and Tukey HSD post hoc test post-test. *P* < 0.05. **Figure S2.** Size distribution of isotigs by the transcriptome sequencing. Length distribution of the sequencing reads after trimming low-quality reads. **Figure S3:** Scatter diagram of log ratios (Fold change) from RT-qPCR data and microarray data of the selected 15 genes in drought tolerant and sensitive plants (4 weeks-old greenhouse-grown) in normal and drought conditions. Regression equation and correlation coefficient (r) are indicated in the diagram. **Figure S4.** Tissue specific gene expression of drought response genes in different vetch accessions. **A.** Expression levels of candidate genes for drought response and/or drought tolerance determined by real-time RT-PCR analysis using GADPH as standard gene for normalization in tolerant and sensitive *Vicia* plants under drought in aerial part of 4 weeks-old greenhouse-grown plants under drought or under watering (control). Values are the average (± standard deviation) of at least three assays. **B.** Same assay than shown in A, but in the radicular part of the plant. Asterisks show differences *p* < 0.05 (Student’s test) referred to control tolerant plants. **Figure S5.** Validation of primers designed for amplification of PCR fragments containing some SNPs (A) or SSRs(B) identified in this work, using the 6 indicated vetch accessions. Amplicon size is arrow indicated. **Table S1.** Summary statistics of *V. sativa* transcriptome sequencing and assembly data. **Table S2.** Gene-specific primers used for quantitative real-time PCR assays. **Table S3.** Gene-specific primers used for validation of SNPs. **Table S4.** Gene-specific primers used for validation of SSRs.

## Data Availability

The datasets generated and/or analyzed during the current study are included in Additional Tables.

## Abbreviations

ABA: Abscisic acid
BP: Biological processes
CC: Cellular components
CFDA: Carboxifluorescein diacetate
Chl: Chlorophyll
COGs: Clusters of Orthologous groups
CRF: Plant Genetic Resources Centre
DEG: Differential expressed genes
DesW: Desiccated weight
DryW: Dry weight
GADPDH: GlycerAldehyde-3-Phosphate-DeHydrogenase
GO: Gene Ontology
KEGG: Kyoto Encyclopedia of Genes and Genomes
KOG: Eukaryotic Orthologous Groups of proteins
LEA: Late embryogenesis abundant
LRR: Leucine-rich repeat
MAS: Marker-assisted selection
MF: Molecular functions
NCBI: National Centre for Biotechnology Information
PEG: Polyethylene glycol
Pfam: Protein family
RNA-Seq: RNA-sequencing
ROS: Reactive Oxygen Species
SNPs: Single nucleotide polymorphisms
SSRs: Simple sequence repeats
UBC: Ubiquitine
WCs: Water content
