# Supplementary material for: Molecular bases for drought tolerance in common vetch: designing new molecular breeding tools
Source: BMC Plant Biol. 2020 Feb 13;20:71. doi: 10.1186/s12870-020-2267-z (PMC7020375; doi:10.1186/s12870-020-2267-z)
Supplement: Supplementary file 3 — Additional file 3 Figure S1. Evaluation of epicuticular wax content, root and aerial weight, osmolyte levels and pigment content for accession identified as drought tolerant (284, 510 and 521 identified with plain colours on the histograms bars of all the figures) and drought sensitives (502, 506 and 545 identified with ornamented wefts on the histogram bars of all the figures). A. Epicuticular wax content from 4-week-old leaves from greenhouse-grown plants. B. Weight of aerial and radicular part of different 4-week-old greenhouse-grown varieties. C-D. Soluble sugars (lower panel) and free proline (upper panel) content were determined on 4-week-old greenhouse-grown plants under control condition or drought treated (3 experiments; n = 10 plants/each). E-F. Pigment content: Anthocyanin levels (E) or chlorophyll a and b levels (F) from 4 week-old greenhouse-grown plants under control condition or drought treated (3 experiments; n = 20 plants/each). A-E. Values are means ± sd. Different letters indicate significantly differences analysed by ANOVA and Tukey HSD post hoc test post-test. P < 0.05. Figure S2. Size distribution of isotigs by the transcriptome sequencing. Length distribution of the sequencing reads after trimming low-quality reads. Figure S3: Scatter diagram of log ratios (Fold change) from RT-qPCR data and microarray data of the selected 15 genes in drought tolerant and sensitive plants (4 weeks-old greenhouse-grown) in normal and drought conditions. Regression equation and correlation coefficient (r) are indicated in the diagram. Figure S4. Tissue specific gene expression of drought response genes in different vetch accessions. A. Expression levels of candidate genes for drought response and/or drought tolerance determined by real-time RT-PCR analysis using GADPH as standard gene for normalization in tolerant and sensitive Vicia plants under drought in aerial part of 4 weeks-old greenhouse-grown plants under drought or under watering (control). Values are t [file 12870_2020_2267_MOESM3_ESM.pptx]

## Slide 1
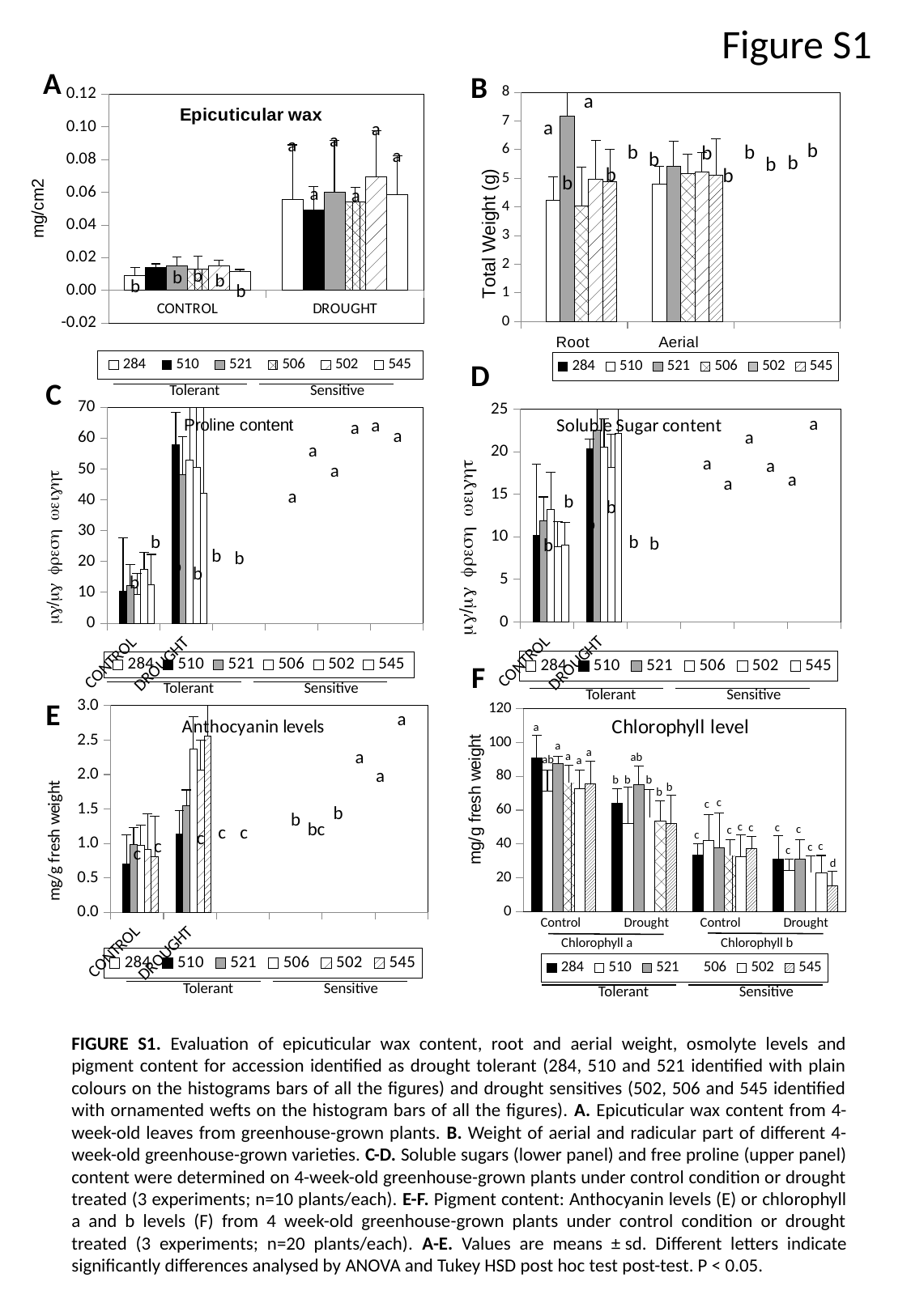

Figure S1
### Chart
| Category | 284 | 510 | 521 | 506 | 502 | 545 |
|---|---|---|---|---|---|---|
| Root | 6.7323 | 4.2388 | 7.163999999999999 | 4.0329 | 4.9569 | 4.8698999999999995 |
| Aerial | 5.441200000000001 | 4.802499999999999 | 5.421399999999999 | 5.1709999999999985 | 5.211 | 5.1209999999999996 |A
B
### Chart: Epicuticular wax
| Category | 284 | 510 | 521 | 506 | 502 | 545 |
|---|---|---|---|---|---|---|
| CONTROL | 0.00915734458104806 | 0.014091714642897485 | 0.014751792732104595 | 0.01286301300355438 | 0.014917126415744577 | 0.011519065336890618 |
| DROUGHT | 0.05537555699414702 | 0.04927505731517254 | 0.06005413788796362 | 0.05398042594403596 | 0.06928323626385643 | 0.05860126162291566 |a
a
a
a
a
a
a
a
b
b
b
b
b
b
b
b
b
b
b
b
b
b
b
b
D
### Chart: Proline content
| Category | 284 | 510 | 521 | 506 | 502 | 545 |
|---|---|---|---|---|---|---|
| CONTROL | 7.969839401612323 | 10.300987170902294 | 12.16685608314639 | 9.33272699607453 | 17.471482495561723 | 12.388747390996036 |
| DROUGHT | 42.7095083266579 | 57.88936168020956 | 48.12227244754884 | 52.877567430266346 | 50.52815804669183 | 42.06706952583615 |C
Tolerant
Sensitive
### Chart: Soluble Sugar content
| Category | 284 | 510 | 521 | 506 | 502 | 545 |
|---|---|---|---|---|---|---|
| CONTROL | 9.238256130642506 | 10.201926848076992 | 11.863282166038648 | 13.254536273383085 | 8.833045278824185 | 9.003532772758888 |
| DROUGHT | 22.330382268957983 | 20.335281251099502 | 22.497049142498454 | 20.49014856328562 | 18.193472902158074 | 22.12202101494561 |a
a
a
a
a
a
a
b
b
b
b
b
b
a
a
a
a
a
b
b
b
b
b
b
F
Tolerant
Sensitive
### Chart: Anthocyanin levels
| Category | 284 | 510 | 521 | 506 | 502 | 545 |
|---|---|---|---|---|---|---|
| CONTROL | 0.8592631827632393 | 0.7034071931051427 | 0.9825730227518958 | 0.9762238938658477 | 0.9148689094387106 | 0.811166762089572 |
| DROUGHT | 1.4297383944422897 | 1.138033947642332 | 1.5448054038362788 | 2.363901535437159 | 2.0647789984752527 | 2.555657982316118 |Tolerant
Sensitive
### Chart: Chlorophyll level
| Category | 284 | 510 | 521 | 506 | 502 | 545 |
|---|---|---|---|---|---|---|
| Chlorophyll a | 90.76373333333332 | 71.40936444444444 | 87.6 | 76.08577142857139 | 72.96797333333333 | 75.45432 |
| Chlorophyll a | 64.0146690459906 | 52.14249856583612 | 75.01073576352954 | 55.54725434866563 | 53.66816009755779 | 52.229569664014164 |
| Chlorophyll b | 33.6134438095238 | 42.09261777777778 | 37.692531047619056 | 33.13545333333333 | 32.37669066666664 | 37.50635295238096 |
| Chlorophyll b | 30.914813064734986 | 24.25724700797988 | 30.91831129860529 | 22.909182724058514 | 23.003650709744534 | 15.482034473488975 |E
a
a
a
a
a
ab
ab
a
b
b
b
b
b
c
c
c
c
c
c
c
c
c
c
c
d
a
a
b
b
bc
c
c
c
c
c
c
Control
Drought
Control
Drought
Chlorophyll b
Chlorophyll a
Tolerant
Sensitive
Tolerant
Sensitive
FIGURE S1. Evaluation of epicuticular wax content, root and aerial weight, osmolyte levels and pigment content for accession identified as drought tolerant (284, 510 and 521 identified with plain colours on the histograms bars of all the figures) and drought sensitives (502, 506 and 545 identified with ornamented wefts on the histogram bars of all the figures). A. Epicuticular wax content from 4-week-old leaves from greenhouse-grown plants. B. Weight of aerial and radicular part of different 4-week-old greenhouse-grown varieties. C-D. Soluble sugars (lower panel) and free proline (upper panel) content were determined on 4-week-old greenhouse-grown plants under control condition or drought treated (3 experiments; n=10 plants/each). E-F. Pigment content: Anthocyanin levels (E) or chlorophyll a and b levels (F) from 4 week-old greenhouse-grown plants under control condition or drought treated (3 experiments; n=20 plants/each). A-E. Values are means ± sd. Different letters indicate significantly differences analysed by ANOVA and Tukey HSD post hoc test post-test. P < 0.05.

## Slide 2
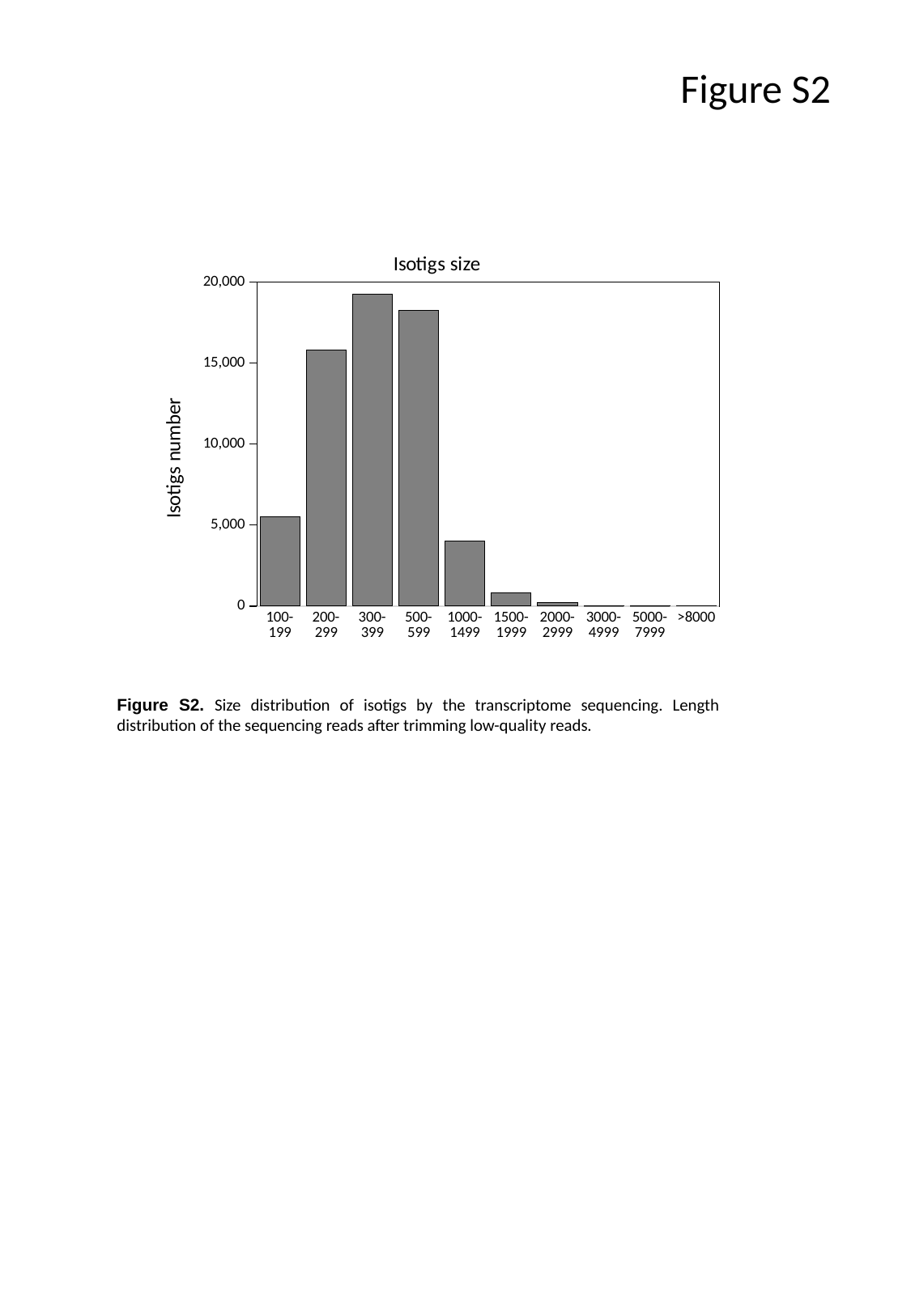

Figure S2
### Chart: Isotigs size
| Category | |
|---|---|
| 100-199 | 5508.0 |
| 200-299 | 15790.0 |
| 300-399 | 19238.0 |
| 500-599 | 18271.0 |
| 1000-1499 | 4000.0 |
| 1500-1999 | 836.0 |
| 2000-2999 | 221.0 |
| 3000-4999 | 13.0 |
| 5000-7999 | 1.0 |
| >8000 | 0.0 |Figure S2. Size distribution of isotigs by the transcriptome sequencing. Length distribution of the sequencing reads after trimming low-quality reads.

## Slide 3
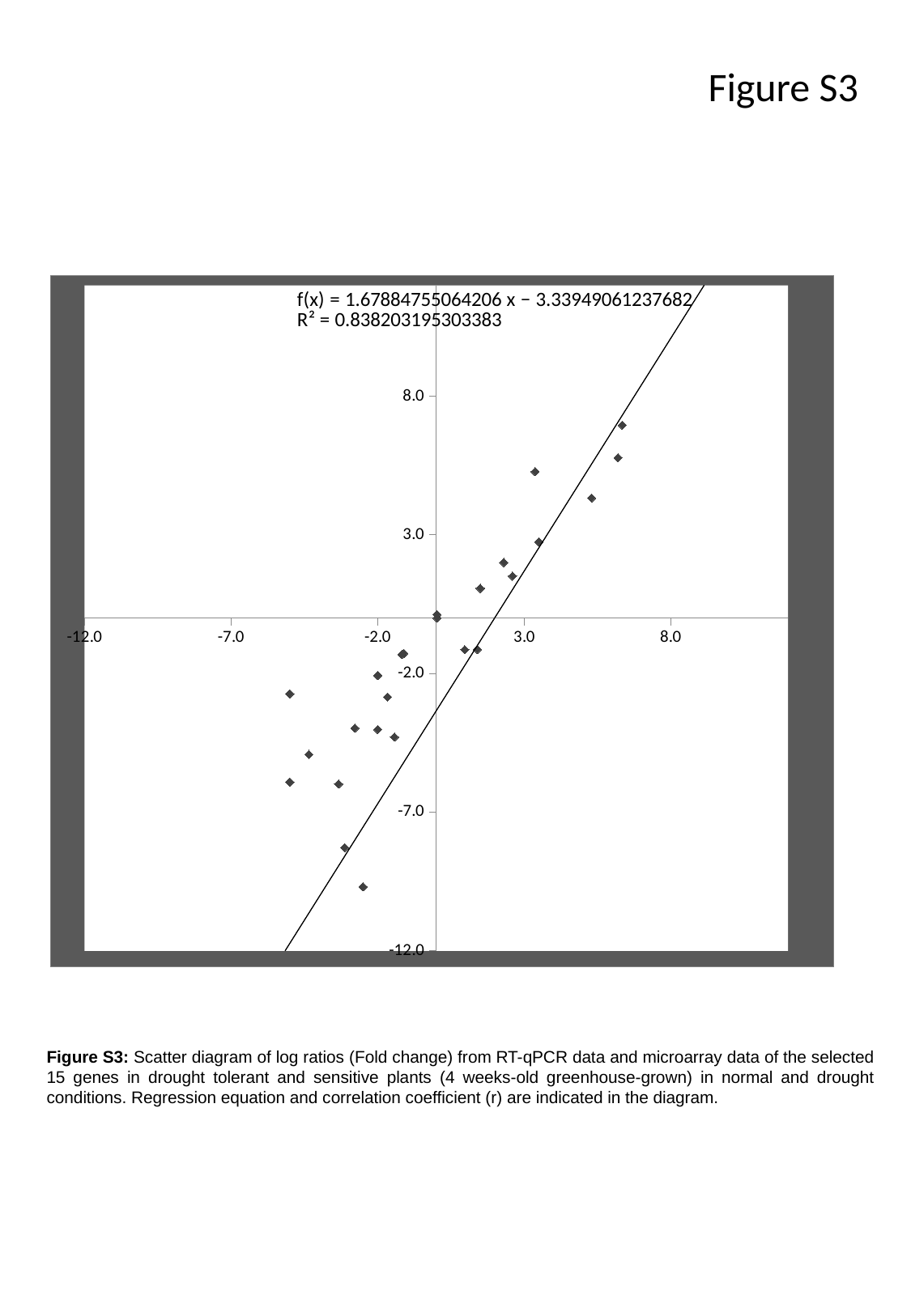

Figure S3
### Chart
| Category | |
|---|---|Figure S3: Scatter diagram of log ratios (Fold change) from RT-qPCR data and microarray data of the selected 15 genes in drought tolerant and sensitive plants (4 weeks-old greenhouse-grown) in normal and drought conditions. Regression equation and correlation coefficient (r) are indicated in the diagram.

## Slide 4
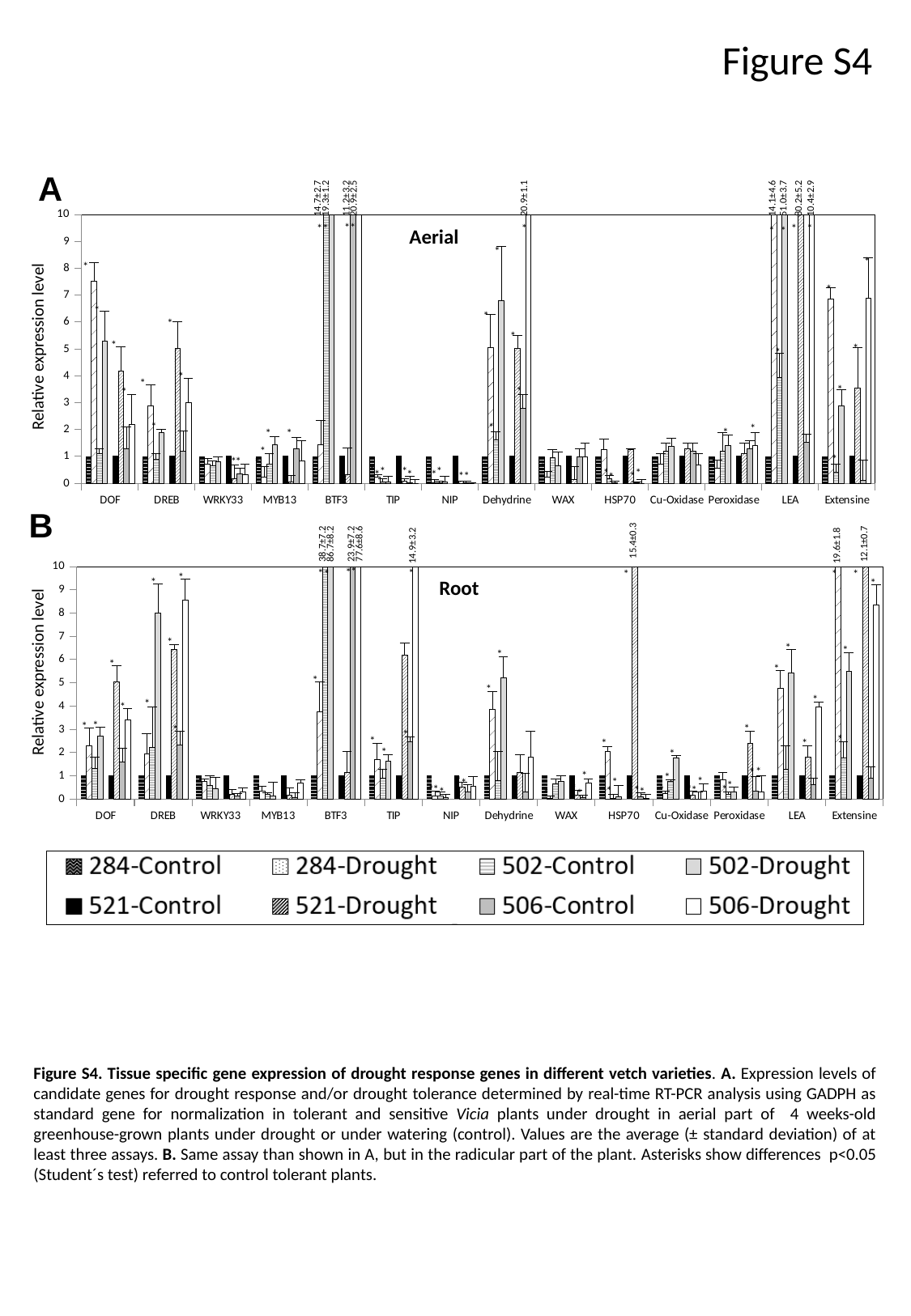

Figure S4
A
14.7±2.7
19.3±1.2
11.2±3.2
20.9±2.5
20.9±1.1
14.1±4.6
51.0±3.7
80.2±5.2
10.4±2.9
### Chart
| Category | 284-Control | 284-Drought | 502-Control | 502-Drought | | 521-Control | 521-Drought | 506-Control | 506-Drought |
|---|---|---|---|---|---|---|---|---|---|
| DOF | 1.0 | 7.516529323094224 | 1.1 | 5.3 | None | 1.0 | 4.190628600836257 | 1.3011614636584155 | 2.2 |
| DREB | 1.0 | 2.8767370035349966 | 0.9122281950547884 | 1.9 | None | 1.0 | 5.01054198505741 | 1.2 | 3.0 |
| WRKY33 | 1.0 | 0.7191842508837492 | 0.6500398189797337 | 0.8 | None | 1.0 | 0.17515045811090732 | 0.35081929425762864 | 0.32632920204706384 |
| MYB13 | 1.0 | 0.22931987984330315 | 0.7043673784903007 | 1.4412960133904333 | None | 1.0 | 0.048141225113193464 | 1.3 | 0.85 |
| BTF3 | 1.0 | 1.4383685017674983 | 14.708722049339055 | 19.259077116877975 | None | 1.0 | 0.3131588740660881 | 11.226217416244111 | 20.9 |
| TIP | 1.0 | 0.2228945633738593 | 0.0654921891900845 | 0.0646129281226196 | None | 1.0 | 0.07475773485907143 | 0.026912122726081653 | 0.00021232570587734876 |
| NIP | 1.0 | 0.029299773724982336 | 0.009380409803218437 | 0.06433584905690837 | None | 1.0 | 0.07390504528831186 | 0.02849494858947824 | 0.0005119770163288243 |
| Dehydrine | 1.0 | 5.067532138430024 | 1.6155215552192204 | 6.809112014369541 | None | 1.0 | 5.01054198505741 | 2.8065543540610287 | 20.885068931012082 |
| WAX | 1.0 | 0.23954744539435796 | 0.9591951044924867 | 0.65891023424287 | None | 1.0 | 0.14025913750135388 | 1.0 | 1.0 |
| HSP70 | 1.0 | 1.2521725094410985 | 0.18523988436424418 | 0.0178450365517309 | None | 1.0 | 1.2581174998379538 | 0.021955101281053775 | 0.00036517273328887106 |
| Cu-Oxidase | 1.0 | 0.7191842508837492 | 1.2 | 1.3826793876824262 | None | 1.0 | 1.3 | 1.2 | 0.7 |
| Peroxidase | 1.0 | 0.5649453942800511 | 1.2 | 1.4 | None | 1.0 | 1.1034464874820193 | 1.3 | 1.4 |
| LEA | 1.0 | 14.07861328522639 | 3.9434271748886074 | 50.97495412121241 | None | 1.0 | 80.16867176091856 | 1.5238279262825072 | 10.44253446550604 |
| Extensine | 1.0 | 6.872015703961167 | 0.4294464052763692 | 2.875407975867096 | None | 1.0 | 3.542988215053999 | 0.10524692589067075 | 6.889503417876903 |Aerial
Relative expression level
B
15.4±0.3
77.6±8.6
38.7±7.2
86.7±8.2
23.9±7.2
12.1±0.7
14.9±3.2
19.6±1.8
### Chart
| Category | 284-Control | 284-Drought | 502-Control | 502-Drought | | 521-Control | 521-Drought | 506-Control | 506-Drought |
|---|---|---|---|---|---|---|---|---|---|
| DOF | 1.0 | 2.3 | 1.3253744379294399 | 2.7 | None | 1.0 | 5.061856703526323 | 1.592102625094635 | 3.4 |
| DREB | 1.0 | 1.964846578533019 | 2.228207105547608 | 8.0013864144813 | None | 1.0 | 6.45790700781228 | 2.3209251557662687 | 8.565079674423517 |
| WRKY33 | 1.0 | 0.770856024587495 | 0.5985526249103575 | 0.4381509827819211 | None | 1.0 | 0.2167844729414779 | 0.12428292448931012 | 0.3 |
| MYB13 | 1.0 | 0.34594949340289616 | 0.1937676991065074 | 0.1426597247272614 | None | 1.0 | 0.1708773795529117 | 0.06332416681104937 | 0.7 |
| BTF3 | 1.0 | 3.7524512607931646 | 38.69298162666758 | 86.69939373497785 | None | 1.0 | 1.1517146626767067 | 23.93997930456073 | 77.5784116602742 |
| TIP | 1.0 | 1.7 | 0.9 | 1.6209616962544282 | None | 1.0 | 6.206729763240698 | 2.4780409011700724 | 14.877222814674576 |
| NIP | 1.0 | 0.15056238935098443 | 0.12094726073251658 | 0.07890989334458613 | None | 1.0 | 0.5244704275476268 | 0.3165049570056604 | 0.5572255569786925 |
| Dehydrine | 1.0 | 3.874965144500473 | 0.7997937985947468 | 5.228329262317696 | None | 1.0 | 1.1480880841109595 | 0.29670476470849827 | 1.8115609339594079 |
| WAX | 1.0 | 0.020139662726301882 | 0.6668804216416537 | 0.7640293684220542 | None | 1.0 | 0.1854282979253669 | 0.07896460847530488 | 0.6860251314473266 |
| HSP70 | 1.0 | 2.063843152539971 | 0.022273930939747645 | 0.09122038061207091 | None | 1.0 | 15.432322934586336 | 0.09119825309389333 | 0.042836467786476096 |
| Cu-Oxidase | 1.0 | 0.2585711701394795 | 0.7797588895057579 | 1.7723333754709698 | None | 1.0 | 0.15668438703172186 | 0.0033293355582445167 | 0.35803768075782055 |
| Peroxidase | 1.0 | 0.8402749223899075 | 0.2 | 0.3 | None | 1.0 | 2.4098332694741935 | 0.3572609164405644 | 0.3 |
| LEA | 1.0 | 4.780903657036435 | 1.2854290660540932 | 5.433945138195766 | None | 1.0 | 1.7982995212549366 | 0.6177093185634643 | 3.979627320671378 |
| Extensine | 1.0 | 19.640140338338494 | 1.7620440607680676 | 5.5 | None | 1.0 | 12.10040418811346 | 0.9 | 8.345701572661781 |Root
Relative expression level
*
*
*
*
*
*
*
*
*
*
*
*
*
*
*
*
*
*
*
*
*
*
*
*
*
*
*
*
*
*
*
*
*
*
*
*
*
*
*
*
*
*
*
*
*
*
*
*
*
*
*
*
*
*
*
*
*
*
*
*
*
*
*
*
*
*
*
*
*
*
*
*
*
*
*
*
*
*
*
*
*
*
*
*
*
*
*
*
*
*
*
*
*
*
*
*
*
*
*
*
*
*
*
*
*
*
*
*
*
*
*
Figure S4. Tissue specific gene expression of drought response genes in different vetch varieties. A. Expression levels of candidate genes for drought response and/or drought tolerance determined by real-time RT-PCR analysis using GADPH as standard gene for normalization in tolerant and sensitive Vicia plants under drought in aerial part of 4 weeks-old greenhouse-grown plants under drought or under watering (control). Values are the average (± standard deviation) of at least three assays. B. Same assay than shown in A, but in the radicular part of the plant. Asterisks show differences p<0.05 (Student´s test) referred to control tolerant plants.

## Slide 5
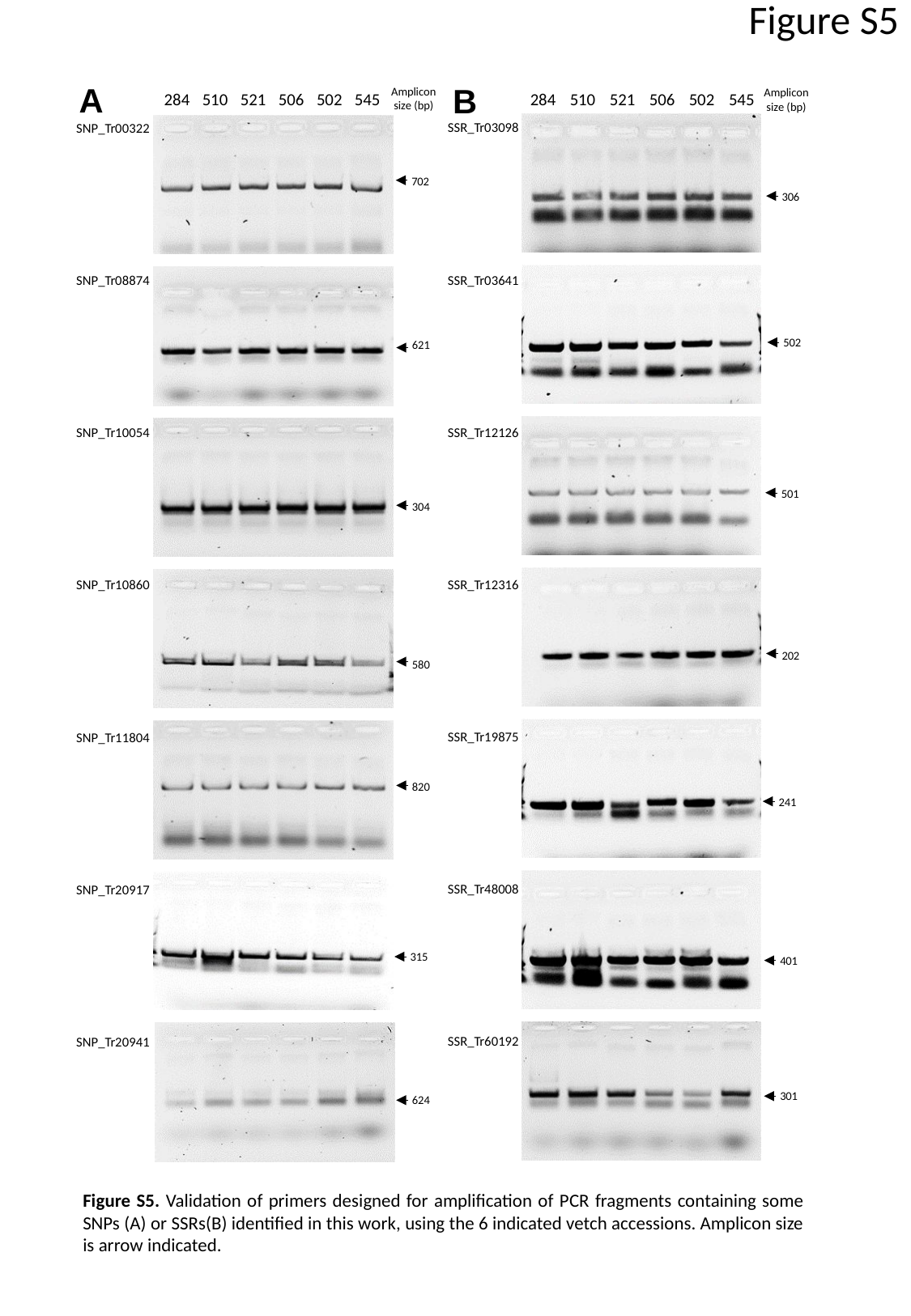

Figure S5
A
B
Amplicon
size (bp)
Amplicon
size (bp)
284
510
521
506
502
545
284
510
521
506
502
545
SSR_Tr03098
SNP_Tr00322
702
306
SSR_Tr03641
SNP_Tr08874
502
621
SSR_Tr12126
SNP_Tr10054
501
304
SSR_Tr12316
SNP_Tr10860
202
580
SSR_Tr19875
SNP_Tr11804
820
241
SSR_Tr48008
SNP_Tr20917
315
401
SSR_Tr60192
SNP_Tr20941
301
624
Figure S5. Validation of primers designed for amplification of PCR fragments containing some SNPs (A) or SSRs(B) identified in this work, using the 6 indicated vetch accessions. Amplicon size is arrow indicated.

## Slide 6
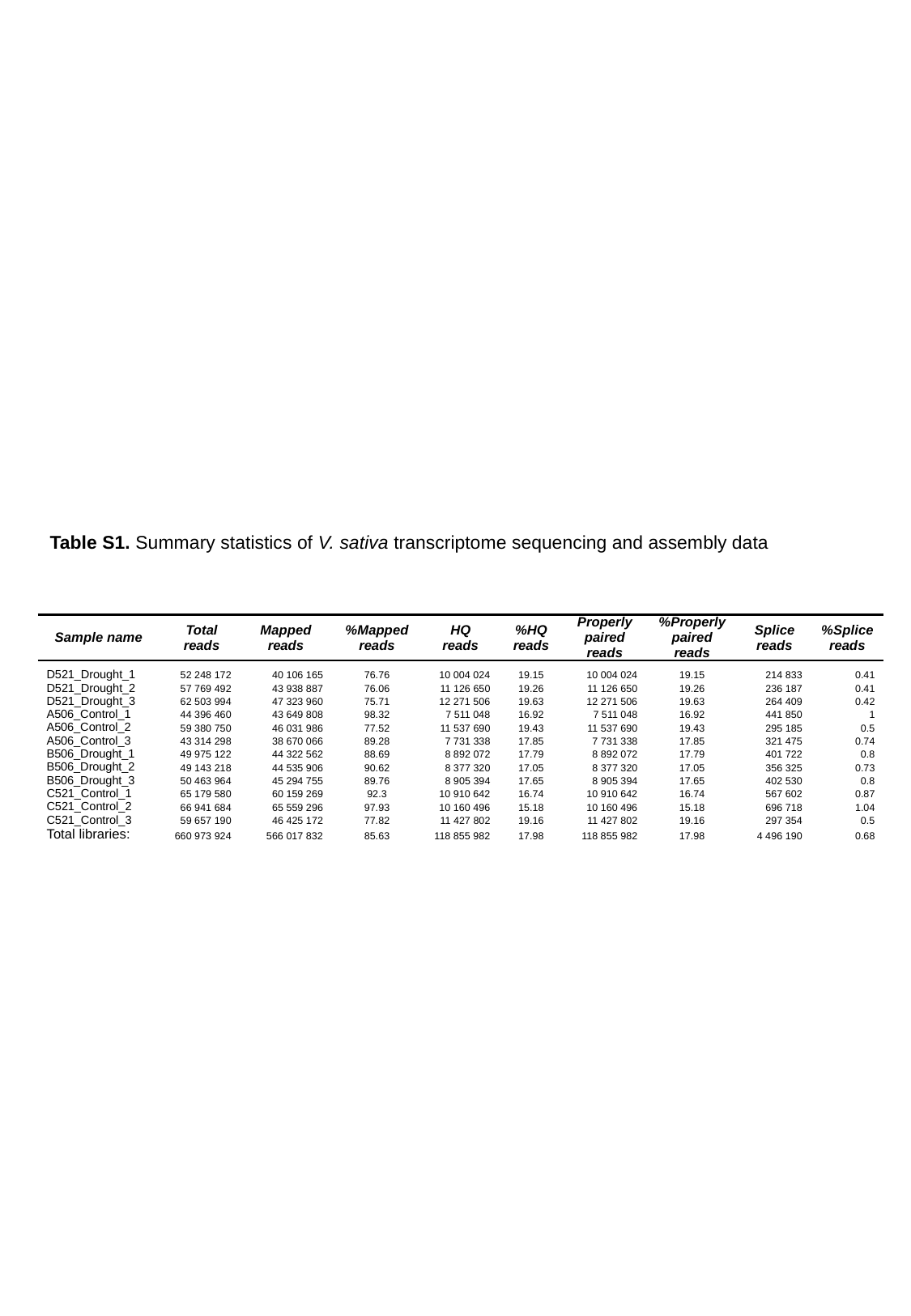

Table S1. Summary statistics of V. sativa transcriptome sequencing and assembly data
| Sample name | Total reads | Mapped reads | %Mapped reads | HQ reads | %HQ reads | Properly paired reads | %Properly paired reads | Splice reads | %Splice reads |
| --- | --- | --- | --- | --- | --- | --- | --- | --- | --- |
| | | | | | | | | | |
| D521\_Drought\_1 | 52 248 172 | 40 106 165 | 76.76 | 10 004 024 | 19.15 | 10 004 024 | 19.15 | 214 833 | 0.41 |
| D521\_Drought\_2 | 57 769 492 | 43 938 887 | 76.06 | 11 126 650 | 19.26 | 11 126 650 | 19.26 | 236 187 | 0.41 |
| D521\_Drought\_3 | 62 503 994 | 47 323 960 | 75.71 | 12 271 506 | 19.63 | 12 271 506 | 19.63 | 264 409 | 0.42 |
| A506\_Control\_1 | 44 396 460 | 43 649 808 | 98.32 | 7 511 048 | 16.92 | 7 511 048 | 16.92 | 441 850 | 1 |
| A506\_Control\_2 | 59 380 750 | 46 031 986 | 77.52 | 11 537 690 | 19.43 | 11 537 690 | 19.43 | 295 185 | 0.5 |
| A506\_Control\_3 | 43 314 298 | 38 670 066 | 89.28 | 7 731 338 | 17.85 | 7 731 338 | 17.85 | 321 475 | 0.74 |
| B506\_Drought\_1 | 49 975 122 | 44 322 562 | 88.69 | 8 892 072 | 17.79 | 8 892 072 | 17.79 | 401 722 | 0.8 |
| B506\_Drought\_2 | 49 143 218 | 44 535 906 | 90.62 | 8 377 320 | 17.05 | 8 377 320 | 17.05 | 356 325 | 0.73 |
| B506\_Drought\_3 | 50 463 964 | 45 294 755 | 89.76 | 8 905 394 | 17.65 | 8 905 394 | 17.65 | 402 530 | 0.8 |
| C521\_Control\_1 | 65 179 580 | 60 159 269 | 92.3 | 10 910 642 | 16.74 | 10 910 642 | 16.74 | 567 602 | 0.87 |
| C521\_Control\_2 | 66 941 684 | 65 559 296 | 97.93 | 10 160 496 | 15.18 | 10 160 496 | 15.18 | 696 718 | 1.04 |
| C521\_Control\_3 | 59 657 190 | 46 425 172 | 77.82 | 11 427 802 | 19.16 | 11 427 802 | 19.16 | 297 354 | 0.5 |
| Total libraries: | 660 973 924 | 566 017 832 | 85.63 | 118 855 982 | 17.98 | 118 855 982 | 17.98 | 4 496 190 | 0.68 |

## Slide 7
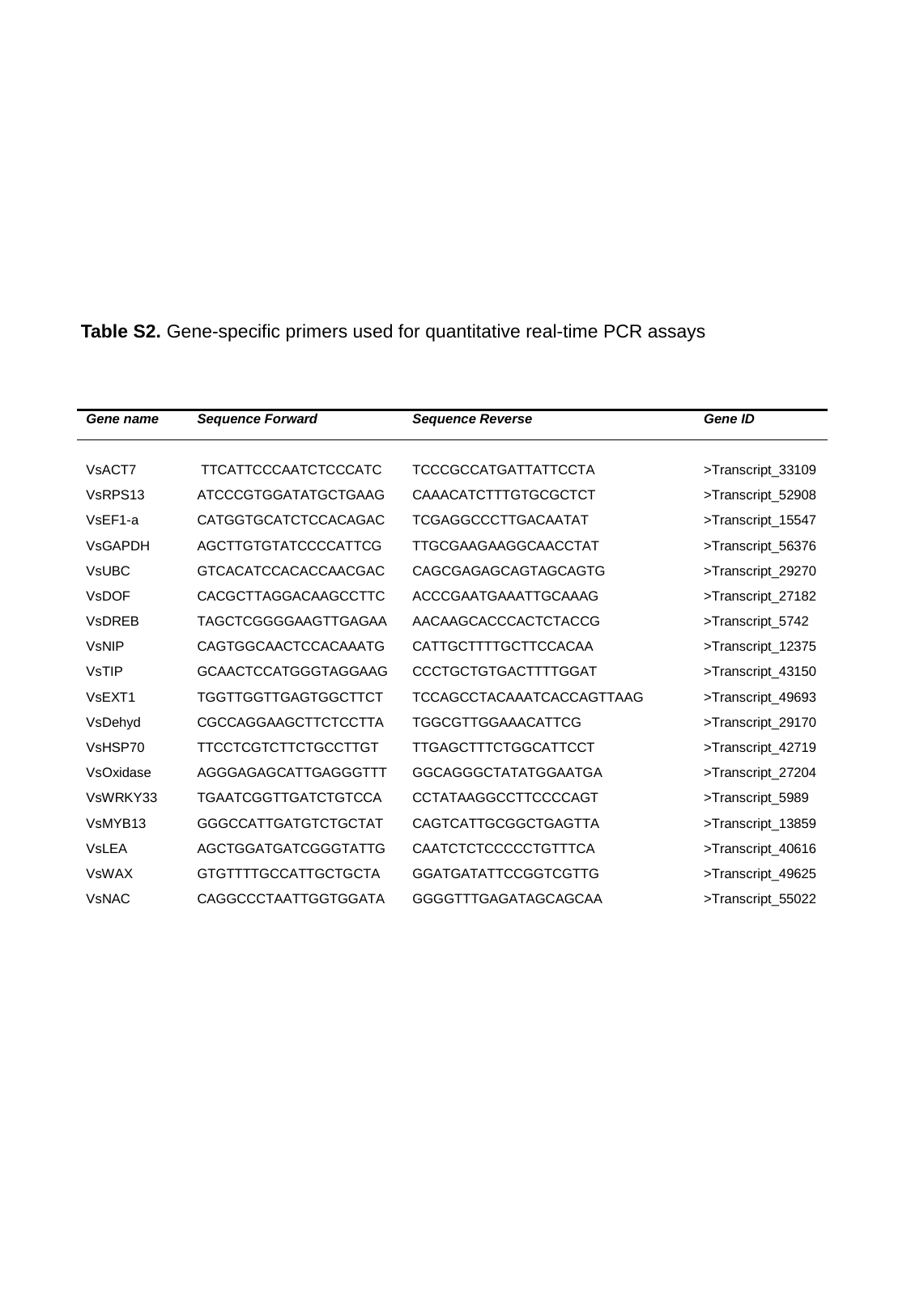

Table S2. Gene-specific primers used for quantitative real-time PCR assays

## Slide 8
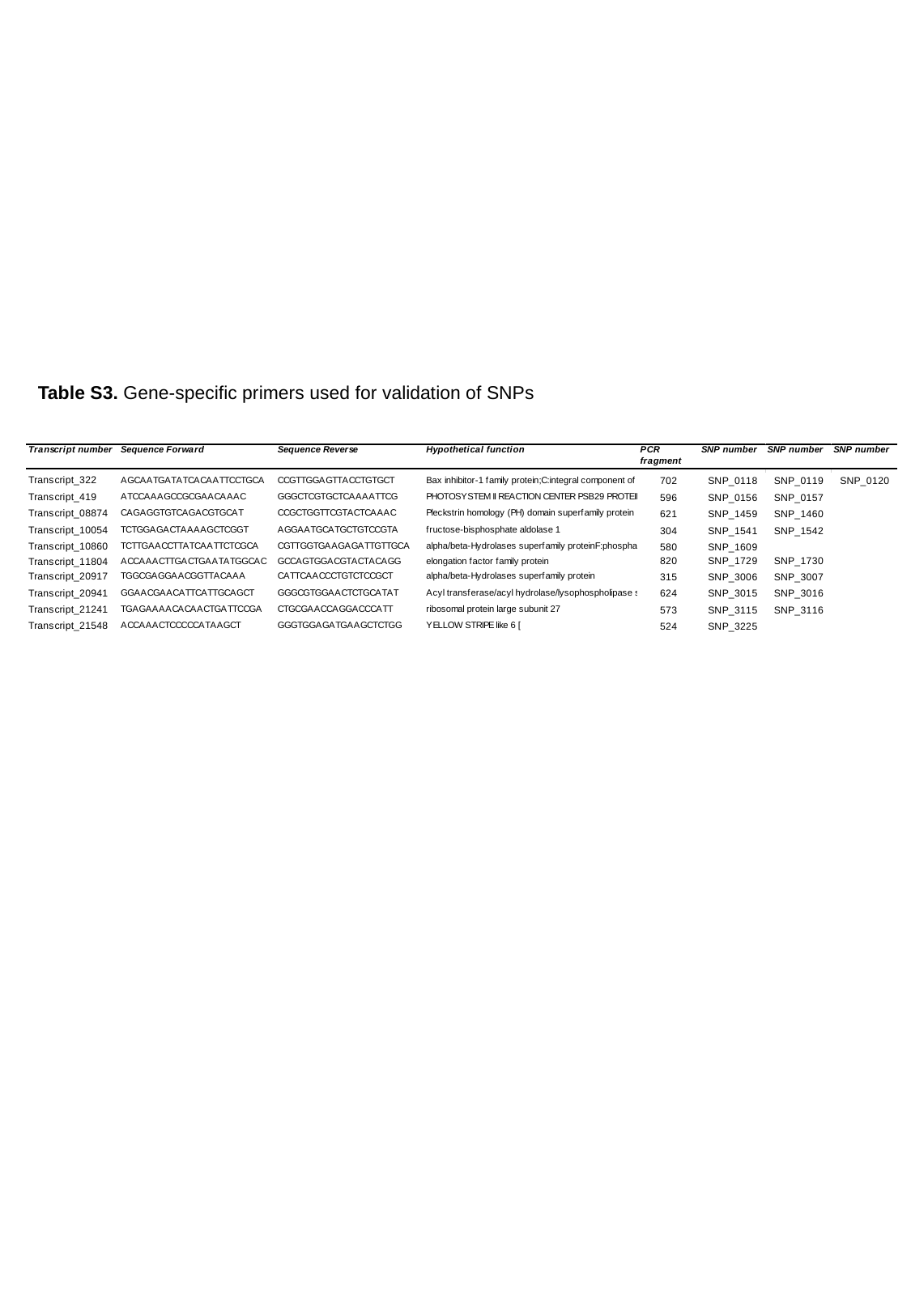

Table S3. Gene-specific primers used for validation of SNPs

## Slide 9
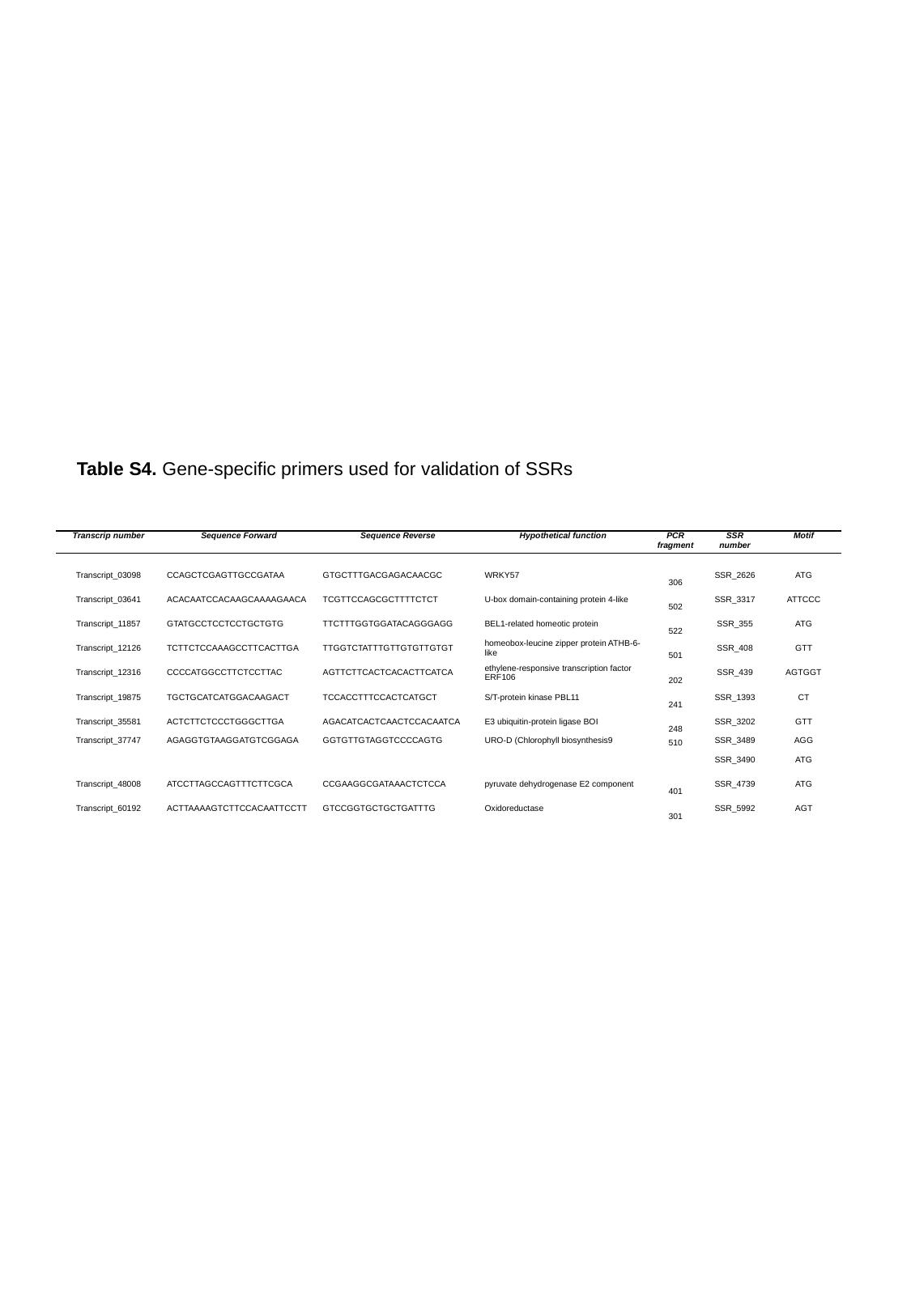

Table S4. Gene-specific primers used for validation of SSRs
| Transcrip number | Sequence Forward | Sequence Reverse | Hypothetical function | PCR fragment | SSR number | Motif |
| --- | --- | --- | --- | --- | --- | --- |
| | | | | | | |
| Transcript\_03098 | CCAGCTCGAGTTGCCGATAA | GTGCTTTGACGAGACAACGC | WRKY57 | 306 | SSR\_2626 | ATG |
| Transcript\_03641 | ACACAATCCACAAGCAAAAGAACA | TCGTTCCAGCGCTTTTCTCT | U-box domain-containing protein 4-like | 502 | SSR\_3317 | ATTCCC |
| Transcript\_11857 | GTATGCCTCCTCCTGCTGTG | TTCTTTGGTGGATACAGGGAGG | BEL1-related homeotic protein | 522 | SSR\_355 | ATG |
| Transcript\_12126 | TCTTCTCCAAAGCCTTCACTTGA | TTGGTCTATTTGTTGTGTTGTGT | homeobox-leucine zipper protein ATHB-6-like | 501 | SSR\_408 | GTT |
| Transcript\_12316 | CCCCATGGCCTTCTCCTTAC | AGTTCTTCACTCACACTTCATCA | ethylene-responsive transcription factor ERF106 | 202 | SSR\_439 | AGTGGT |
| Transcript\_19875 | TGCTGCATCATGGACAAGACT | TCCACCTTTCCACTCATGCT | S/T-protein kinase PBL11 | 241 | SSR\_1393 | CT |
| Transcript\_35581 | ACTCTTCTCCCTGGGCTTGA | AGACATCACTCAACTCCACAATCA | E3 ubiquitin-protein ligase BOI | 248 | SSR\_3202 | GTT |
| Transcript\_37747 | AGAGGTGTAAGGATGTCGGAGA | GGTGTTGTAGGTCCCCAGTG | URO-D (Chlorophyll biosynthesis9 | 510 | SSR\_3489 | AGG |
| | | | | | SSR\_3490 | ATG |
| Transcript\_48008 | ATCCTTAGCCAGTTTCTTCGCA | CCGAAGGCGATAAACTCTCCA | pyruvate dehydrogenase E2 component | 401 | SSR\_4739 | ATG |
| Transcript\_60192 | ACTTAAAAGTCTTCCACAATTCCTT | GTCCGGTGCTGCTGATTTG | Oxidoreductase | 301 | SSR\_5992 | AGT |
